# A Critical Evaluation of Network and Pathway-Based Classifiers for Outcome Prediction in Breast Cancer

**DOI:** 10.1371/journal.pone.0034796

**Published:** 2012-04-27

**Authors:** Christine Staiger, Sidney Cadot, Raul Kooter, Marcus Dittrich, Tobias Müller, Gunnar W. Klau, Lodewyk F. A. Wessels

**Affiliations:** 1 Centrum Wiskunde & Informatica, Life Sciences Group, The Netherlands; 2 Bioinformatics and Statistics, The Netherlands Cancer Institute, Amsterdam, The Netherlands; 3 Delft Bioinformatics Lab, Faculty of Electrical Engineering, Mathematics and Computer Science, Delft, The Netherlands; 4 Department of Bioinformatics, Biocenter, University of Würzburg, Würzburg, Germany; 5 Netherlands Institute for Systems Biology, Amsterdam, The Netherlands; 6 Cancer Systems Biology Center, The Netherlands Cancer Institute, Amsterdam, The Netherlands; Centro de Investigación Príncipe Felipe, Spain

## Abstract

Recently, several classifiers that combine primary tumor data, like gene expression data, and secondary data sources, such as protein-protein interaction networks, have been proposed for predicting outcome in breast cancer. In these approaches, new composite features are typically constructed by aggregating the expression levels of several genes. The secondary data sources are employed to guide this aggregation. Although many studies claim that these approaches improve classification performance over single genes classifiers, the gain in performance is difficult to assess. This stems mainly from the fact that different breast cancer data sets and validation procedures are employed to assess the performance. Here we address these issues by employing a large cohort of six breast cancer data sets as benchmark set and by performing an unbiased evaluation of the classification accuracies of the different approaches. Contrary to previous claims, we find that composite feature classifiers do not outperform simple single genes classifiers. We investigate the effect of (1) the number of selected features; (2) the specific gene set from which features are selected; (3) the size of the training set and (4) the heterogeneity of the data set on the performance of composite feature and single genes classifiers. Strikingly, we find that randomization of secondary data sources, which destroys all biological information in these sources, does not result in a deterioration in performance of composite feature classifiers. Finally, we show that when a proper correction for gene set size is performed, the stability of single genes sets is similar to the stability of composite feature sets. Based on these results there is currently no reason to prefer prognostic classifiers based on composite features over single genes classifiers for predicting outcome in breast cancer.

## Introduction

Modern high-throughput methods provide the means to observe genome wide changes in gene expression patterns in breast cancer samples. Gene expression signatures have been proposed [1], [2] to predict prognosis in breast cancer patients, but were shown to vary substantially between data sets. One possible explanation for this effect is that the data sets on which the predictors are trained are typically poorly dimensioned, consisting of many more genes than samples. Integrating secondary data sources like protein-protein interaction (PPI) networks, co-expression networks or pathways from databases such as KEGG, has recently been proposed to overcome variability of prognostic signatures and to increase their prognostic performance [3]–[7]. Many of these studies claim that combining gene expression data with secondary data sources to construct composite features results in higher accuracy in outcome prediction and higher stability of the obtained signatures. In addition, inclusion of the secondary sources raises the hope that the obtained signatures will be more interpretable and thus provide more insight into the molecular mechanisms governing survival in breast cancer.

The underlying idea of these methods is that genes do not act in isolation, and that complex diseases such as cancer are actually caused by the deregulation of complete processes or pathways, representing ‘hallmarks of cancer’ [8]. This is unlikely to happen due to an aberration in a single gene, and often multiple genes need to be perturbed to disable a process. This leads to the notion that aggregating gene expression of functionally linked genes smooths out noise and provides more power to detect deregulation of complete functional units and hence to obtain a clearer picture of the biological process underlying tumorigenesis and disease outcome.

The observed improvement in classification accuracy achieved by the approaches employing secondary data is hard to assess since it is dependent on many factors such as the specific data sets and evaluation protocol employed. To shed more light on this issue we performed an extensive comparison of a simple, single genes based classifier with three of the most popular approaches that include secondary data sources in the construction of the classifier. More specifically, we included the approaches proposed by Chuang *et al.* [3], Lee *et al.* [4] and Taylor *et al.* [5]. We investigated how these methods perform with respect to classification accuracy and stability of the set of features included in the classifiers. We will now briefly outline how the approaches work and point out some of the claims made by the authors. Detailed descriptions are provided in the Methods section.

Chuang *et al.* [3] describe a greedy search algorithm on PPI networks. For each defined subnetwork, a composite feature is defined as a variant of the average of the expression values of the genes included in the subnetwork. The score that guides the search is the association of the composite feature with patient outcome. Significance testing and a feature selection step are employed to select the set of composite features used in the final classifier. The authors claim that classification based on subnetwork markers improves prediction performance on two breast cancer data sets. Moreover, they state that subnetwork markers are more reproducible across different breast cancer studies than single genes markers.

Lee *et al.* [4] employ gene sets from the MsigDB database [9] as secondary data source. The association of the composite feature with patient outcome is used as performance criterion, and a greedy search is employed to select a subset of genes from a gene set to constitute the composite feature. The value of the composite feature is derived from the expression values of the subset of genes as defined in Chuang *et al.* [3]. In contrast to Chuang *et al.*, Lee *et al.* do not exploit the connectivity of the pathway in the construction of the composite features. Lee *et al.* claim that by using these pathway activities a higher classification performance can be achieved on different cancer types, most notably leukemia, lung and breast cancer. They also report a higher overlap between genes in the top scoring composite features compared to the top scoring single genes.

The underlying assumption in the study by Taylor *et al.* [5] is that disease-causing perturbations disturb the organization of the interactome, which then has an effect on outcome. They concentrate on highly connected proteins, so-called hubs, as these proteins act as organizers in the molecular network. In contrast to Lee *et al.* and Chuang *et al.*, Taylor *et al.* detect aberrations in the correlation structure between a hub and its immediate interactors. As correlation between two genes cannot be assessed for a single sample, Taylor *et al.* employ the pairwise expression difference between the hub and each of its interactors as features for the classifier. While no claims are made regarding performance improvements, we included this approach in the comparison as it is a recently proposed, novel approach for exploiting secondary data sources to predict outcome in breast cancer.

Table 1 provides a summary of the characteristics of all methods included in the comparison. It lists a description of each approach, the secondary data sources employed, the types of (composite) features and how the value of a (composite) feature is computed for a single tumor.

**Table 1:** Overview of evaluated feature extraction methods.

Method	Symbol	Description	Secondary data	Feature	Feature value
Single genes	SG	Calculates t-statistic between mRNA expression distributions of the two patient groups	None	Single gene	mRNA expression of the gene
Lee*et al.* [4]	L, *Lee*	Calculates for each pathway a set of genes with high t-statistic between the averaged gene expression and the two patient groups	MsigDB, KEGG	Subset of genes in a pathway	Averaged mRNA expression of genes in set
Chuang *et al.* [3]	C, *Chuang*	Calculates subnetworks with high mutual information between the averaged gene expression of the genes in the subnetworks and the class labels of the two patient groups	KEGG, HPRD, I2D, NetC	Genes in a subnetwork	Averaged mRNA expression of the genes in the network
Taylor*et al.* [5]	T, *Taylor*	Finds hub proteins that, given the two patient groups, show different Pearson correlation of the mRNA expression between the hub proteins and all of their direct interactors	KEGG, HPRD, I2D, NetC	Edge between a huband its interactor	Difference of mRNA expression of hub and its interactors (edge weights)

As secondary data sources we used the KEGG database [21] and the C2 data set of the MsigDB [9], as PPI data we used the information from KEGG, HPRD [24] and the OPHID/I2D databases [25]. In addition we used the PPI network published by Chuang *et al.* [3] (NetC).

**Figure 1:**
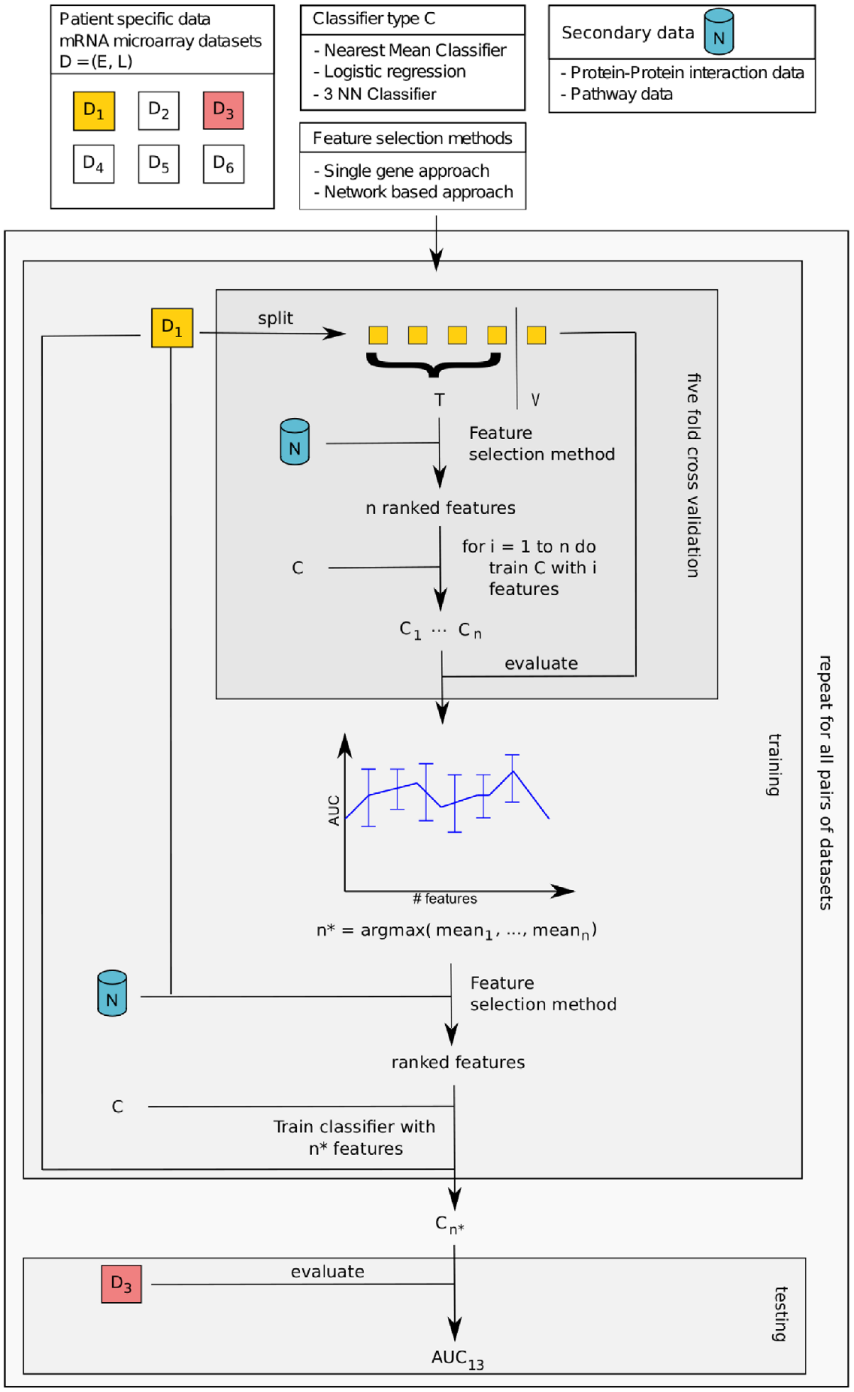
Overview of validation procedure. Input: patient-specific mRNA expression data 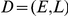 where *E* is an expression matrix and *L* a vector with class labels; an untrained classifier; a feature extraction method and an additional secondary data source *N* is used in the feature extraction method. Five fold cross-validation is used to determine the best performing number of features in the final classifier. The data set 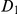 is split into five parts of which four parts are used as training set *T* and one part is used as test or validation set *V*. First *T* is used to extract features and to rank them. A series of classifiers is then trained, where the number of features is gradually increased by adding features according to the ranking. All classifiers are evaluated using *V*. As performance measure the area under the curve (AUC) is used. At the end of the cross-validation each of the five splits has been used for validation once, thus we obtain five different AUC values. We choose the number of features 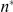 in the final classifier to be the number of features corresponding to the highest mean AUC value. The best 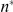 features are determined on the whole data set 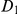 and the final classifier with these features is also trained on 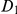. This classifier is then tested on a different data set 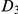. We evaluated all possible pairs of data sets 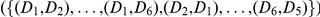 to evaluate each feature extraction method in a certain setting resulting in 30 AUC values for each setting. These values were then analyzed in a box plot or a winchart. The features in the six classifiers (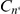 of each data set) were used to determine the stability of gene markers across different data sets.

All three studies listed above use their own specific cross-validation (CV) protocol and evaluate their method on different (combinations of) data sets. This makes it hard to assess the improvement over other methods. In this work, we therefore employ an unbiased training and validation protocol and present a comprehensive evaluation of cross data set classification performance and stability on six publicly available breast cancer data sets. Given that these classifiers are intended to predict the unknown outcome of a patient, we suggest a cross-validation procedure that does not assume any knowledge about the samples used for testing. Thus, we strictly separate the training data set from the test data set, *i.e.* composite feature construction, the selection of the optimized number of features for classification and the training of the final classifier are all performed on the training data set, while the testing of this trained classifier is performed on a completely separate test set without calibrating the classifier on the test data. See Figure 1 and Algorithm 0 for details. In other words, in contrast to previous studies, we strictly distinguish between training and test data.

To prevent biases associated with a specific secondary data source, we tested the algorithms on different types of secondary data sources. (See the Materials and Methods section for detailed descriptions of all these data sources.) We also used three different classifier types, the nearest mean classifier (NMC), logistic regression (LOG) and the 3 nearest neighbor classifier (3NN) to evaluate the influence of the classifier on prediction performance. We chose the NMC and LOG since Popovici *et al.* [10] confirmed earlier findings that these classifiers performed best on various breast cancer related classification tasks. The 3NN is included as an example for a non-linear classifier. Similarly, different feature extraction strategies were employed. While the included set of feature extraction approaches is by no means exhaustive, we employed approaches that were shown to perform well on gene expression based diagnostic problems [11]. All evaluations were performed on a curated collection consisting of six breast cancer cohorts [12] including the cohort from the Netherlands Cancer Institute [13].

In contrast to earlier findings we find that when we apply a proper correction for the number of genes appearing in the composite features, the stability of single genes feature sets is comparable to the stability of composite feature sets. Much to our surprise, and in contrast to other studies, we also find that integrating secondary data, as done in the evaluated methods, does *not* lead to increased classification accuracy when compared to simple single genes based methods. Our findings are partly consistent with the findings of Abraham *et al.* [7], where the authors show that averaging over gene sets does not increase the prediction performance over a single genes classifier.

We investigated several possible factors that may explain the disappointing performance of approaches incorporating secondary data. First, we looked into the effect of the way the number of features is selected. Second, we looked into the effect of the exact size and composition of the starting gene set. This factor could play a role since not all genes are included in secondary data sources, hence classifiers employing secondary data sources may be at a disadvantage compared to single genes classifiers that select the gene set from all genes on the chip. Third, we investigated the effect of sample size. Finally, since breast cancer is a collection of genetically different diseases, heterogeneity in the microarray studies might decrease the classification performance. We therefore looked into the effect of heterogeneity of the data sets on classifier performances. We find that none of the investigated factors change our general findings.

In addition to all these technical factors, we also investigated whether the biological information captured in the secondary data contributes to the classification performance of the composite feature classifiers. To our astonishment we found that composite classifiers constructed from 25 *randomized* versions of the secondary data sources performed comparably to composite classifiers trained on the original, non-randomized data.

We conclude that further research has to be done on finding effective ways to integrate secondary data sources in predictors of outcome in breast cancer. In order to facilitate this research, and to ensure a standardized and objective way of establishing improvements over state-of-the-art approaches, we make all the code, data sets and results employed in this comparison available for download at http://bioinformatics.nki.nl/staiger/software.php.

## Results

### Current Composite Feature Classifiers do not Outperform Single Genes Classifiers on Six Breast Cancer Data Sets

**Figure 2:**
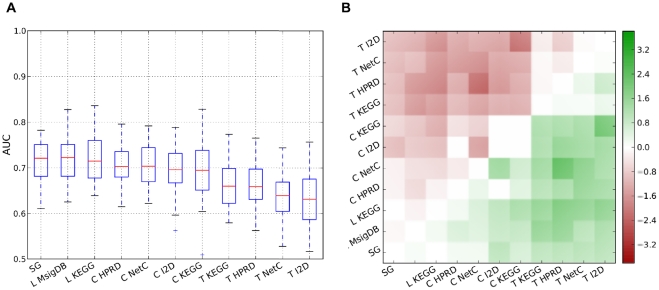
Performance of the NMC employing single genes and composite features constructed from different secondary data sources. For each combination of feature extraction method and secondary data source and each pair of data sets we obtained one AUC value resulting in 30 AUC values per combination. The number of features for each classifier was determined in the cross-validation procedure (CV-optimized). **A:** Each box plot shows the median, the 25% and 75% percentiles and the standard deviation of the 30 AUC values. Outliers are depicted by crosses. The boxes are sorted in descending order according to the median. **B:** This panel shows the result of pairwise comparisons between all combinations of feature extraction methods and secondary data sources. If, for a given combination of training and test data set, the AUC value of classifier *i* is higher (lower) than the AUC value of classifier *j* on the same test data set, it is counted as a win (loss) for classifier *i*. Element (*i*, *j*) in the matrix represents the 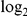 ratio of wins to losses of method *i* compared to method *j*. Green indicates an overall win, red an overall loss and white represents draws. The rows and columns are sorted as in Panel A. **Abbreviations:** SG: Single genes; C: *Chuang*; L: *Lee* and T: *Taylor*.

We compared the performance of a nearest-mean classifier (NMC) using single genes with an NMC employing feature extraction methods based on pathway and PPI data. The results are depicted in Figure 2. For each combination of secondary data source and feature extraction approach, Figure 2A shows the box plots of the area under the receiver-operating characteristics curve (AUC) values obtained for each pair of data sets–using one data set of the pair as training set and the other data set in the pair as test set. The feature extraction approaches are ranked in descending order based on the median AUC values. The box plots suggest that no composite classifier performs better than the single genes classifier. Indeed, testing whether the mean performance of the single genes classifier is different from the mean performance of any composite classifier reveals that there is no difference (null hypothesis can not be rejected) except for *Taylor* and *Chuang* -I2D, where the single genes classifier is clearly superior. See Table 1 in File S1 for details. This fact is confirmed by the pairwise comparisons between all classifiers, see Figure 2B. A green square means that the combination in the row won more frequently over the combination in the column across the data set pairs. The good performance of the single genes classifier is reflected by the fact that the bottom row does not contain a single red box. Also, the generally poor performance of *Taylor* is clearly reflected in the dark red rows associated with this approach.

**Figure 3:**
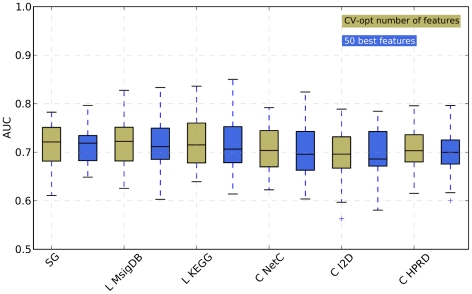
Performance of all classifiers restricted to 50 features. Comparison of the performance of the classifiers when the number of features is trained in the CV procedure (denoted as ‘CV-opt number of features’, same values as in Figure 2) and when the 50 best scoring features (denoted as ‘50 best features’) are selected for classification. We cannot show the values for *Chuang* -KEGG, *Taylor* -KEGG or *Taylor* -HPRD since for some data sets, the number of significant composite features was lower than 50. Abbreviations of methods as in Figure 2.

We also provide the classification results for the logistic regression (LOG) classifiers in Figure 1 in File S1 and Table 3 in File S1 and results for a 3-nearest neighbor (3NN) classifier in Figure 2 in File S1 and Table 5 in File S1. In general, the performances for both classifiers are lower than for the NMC, with the best combination not even reaching an AUC of 0.7 while several NMC classifiers clearly exceed 0.7. For the 3NN classifiers the mean performance of the single genes classifier is not different from the mean performance of any composite classifier. Apart from *Lee* all composite LOG classifiers perform equally or even worse than the single genes LOG classifier. However, it should be noted that the performance of the LOG classifier is highly variable as a function of the number of included features–see Figures 3–5 in File S1. In addition, the training procedure does not converge for all feature values as is evident in the AUC vs. number of features curves that end abruptly. The high sensitivity to the number of features is most evident for the *Taylor* composite NMCs. Clearly, the LOG classifier as employed here (and as employed by Chuang *et al.* ) requires additional regularization to ensure convergence across the whole range of feature values. Also in combination with this classifier, *Taylor* performed poorly. This together with the high computational burden associated with this method, prompted us to omit *Taylor* from the remaining analyses.

**Figure 4 pone-0034796-g004:**
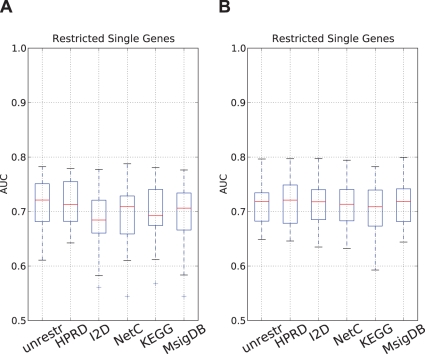
Comparison of single genes classifiers restricted to genes occurring in the secondary data sources. We compared the performance of the single genes classifier trained on all genes present on the microarray ( *unrestr.*) with the performance of single genes classifiers that only employ genes present in the secondary data sources. The resulting classifiers are indicated by the secondary data source whose gene set was employed to train the classifier. **A:** The number of single genes was determined during the cross-validation procedure; **B:** the 50 best scoring single genes were employed.

**Figure 5:**
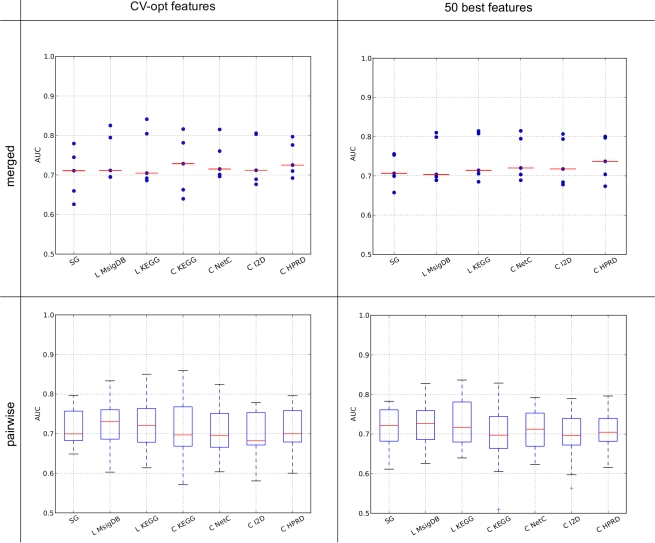
Classification results for merged and paired setting. In the merged setting one Affymetrix data set is set aside as test and the remaining four Affymetrix data sets are merged into a single data set. This is repeated until every one of the five data sets acted as a test set. **Top row:** Results for the merged setting. The red lines indicate the median. **Bottom row:** Only the five Affymetrix data sets were used in the paired setting.

Based on the results presented in Figure 2, we conclude that on the six breast cancer data sets employed in this comparison, composite classifiers employing secondary data sources do not outperform single genes classifiers on the task of predicting outcome in breast cancer, provided that a robust single genes classifier is employed.

### Four Hypotheses Regarding the Lack of Observed Performance Differences

Next we formulated a number of hypotheses that could explain why classifiers employing secondary data sources do not outperform single genes classifiers. These hypotheses relate to (1) the feature selection approach employed; (2) the starting set of genes employed in each of the approaches; (3) the effect of the training set size on performance and (4) the homogeneity of the data set employed. In the following sections we will investigate these hypotheses one by one.

#### The number of selected features does not affect relative performances

**Figure 6:**
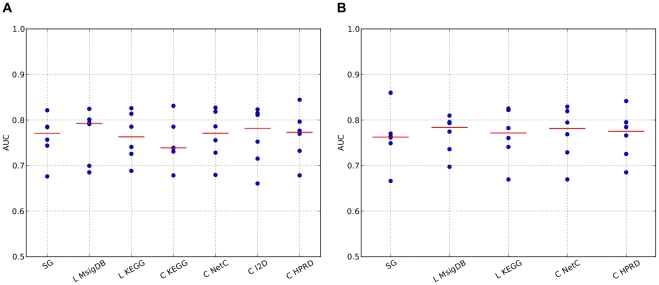
Classification results of the ER positive data only. The ER positive cases from a single data set were set aside as test set while ER positive cases from the remaining five data sets were merged into a single training set. This was repeated until each data set was employed as left-out test set, resulting in six AUC values. The red lines indicate the median. **A**: CV-optimized number of features; **B**: 50 best features.

**Figure 7:**
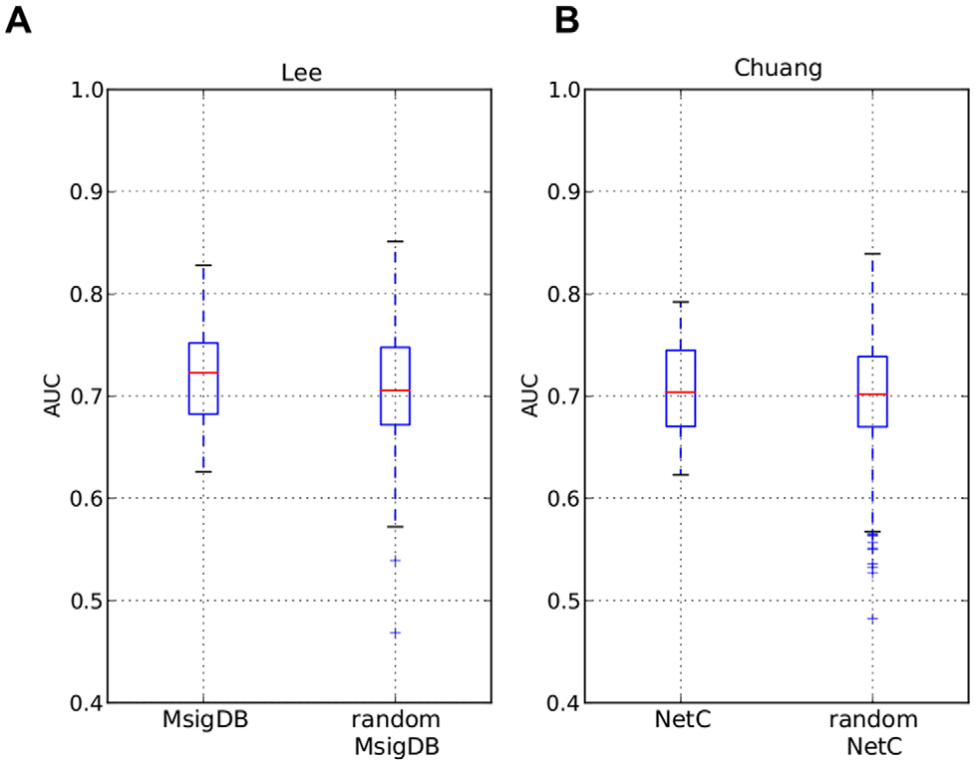
The effect of randomized secondary data sources. **Left:** AUC values obtained with the feature extraction method *Lee* on real and randomized MsigDB pathways. **Right:** AUC values obtained with the feature extraction method *Chuang* on real and randomized PPI networks.

Our cross-validation protocol employs individual feature filtering to select an optimized number of features for the classifier. We (Wessels *et al.* [11]) and others have shown that these simple approaches perform the best in predicting phenotypes based on gene expression data. However, we observed in the curves showing the AUC values as a function of the number of ranked features included in the classifier (Figures 3–5 in File S1) that the AUC values for the NMC are very stable across a large range of features for most approaches. This implies that the absolute maximal AUC value chosen during the feature selection routine might only marginally differ from the performance obtained with other feature values. For this reason, and since the selection of the optimized number of features introduces additional variability between the approaches, we decided to fix the number of features to 50, 100 and 150 for most approaches. We chose these values as they covered the feature ranges across which the performance remained stable in all approaches. The results for fixing the number of features to 50 are depicted in Figure 3 while results for 100 and 150 features are presented in Figures 6 and 7 in File S1. Wilcoxon rank tests show that no classifier using a fixed number of features performs significantly different from its counterpart using the number of features determined by cross-validation. See p-values of the pairwise Wilcoxon rank test in Tables 7, 9 and 11 in File S1. As expected, there are only minor differences between the performance of classifiers when the number of features is restricted to 50, 100 and 150 (Tables 6, 8 and 10 in File S1) with any significant differences favoring single genes. This confirms that the number of features is not a critical parameter. Based on these results, we can conclude that the number of selected features does not explain the observed differences between composite feature classifiers and single genes classifiers.

#### Restricted gene sets are not detrimental to composite feature classifiers

**Figure 8:**
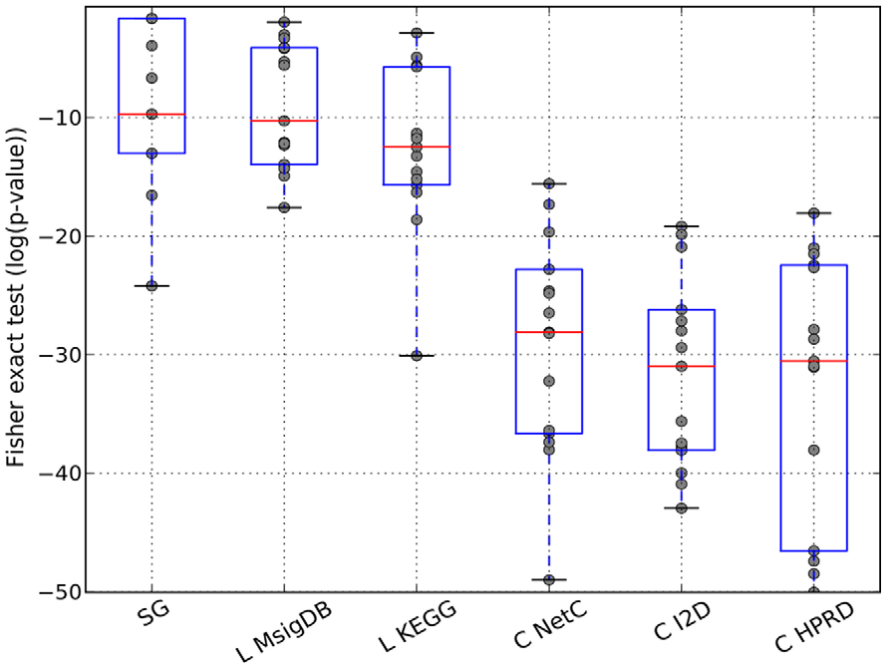
Feature stability when the top 50 features are selected. For each method the Fisher exact test was employed to compute a p-value representing the overlap between the gene sets extracted from two different data sets. This was repeated for all pairwise combinations of data sets, resulting in 15 values. It should be noted that we do not employ the Fisher exact test as a hypothesis test. Instead we interpret the p-values as a measure of overlap between two sets of marker genes. The figure shows the box plots for each method and the individual values (grey dots).

We next hypothesized that the lack of difference in the performance between composite classifiers and single genes classifiers could be caused by the fact that the composite features are bound to the genes annotated in the secondary data while single genes classifiers can employ all genes on the microarray. To test this hypothesis we retrained the single genes classifier, but restricted the set of genes from which features for the classifier could be selected to the genes that are present in the respective secondary data sources. The resulting classifiers are denoted by the secondary data source from which the gene set is derived, while the single genes classifier employing features from the whole microarray is denoted by *unrestr*. The results of this analysis are depicted in Figure 4A. There is significant difference in the performance of the classifiers employing genes annotated in the I2D, KEGG and MsigDB (Table 12 in File S1). However, when accounting for multiple testing only the difference between *unrestr* and I2D remains significant. Moreover, as indicated earlier, the optimization of the number of features by cross-validation introduces significant variation in the number of features without resulting in large performance changes. To eliminate this source of variation from the comparison, we fixed, as before, the number of features to 50, 100 and 150 and repeated the comparisons. The results are depicted in Figure 4B, and also in Figure 8 in File S1 and Tables 13–15 in File S1. We can only find significant differences between the unrestricted single genes classifier and KEGG when the 50 best features are selected and the I2D when employing the 150 best features. However, both of these differences disappear when multiple testing correction is performed. We therefore conclude that the starting gene set has a minor influence on the single genes classifiers. Hence we can reject the hypothesis that feature extraction approaches employing secondary data sources are put at a disadvantage since they can not exploit the full set of genes present on the array.

#### Training set size has no significant effect on performance differences

A third possible factor that could explain the lack of performance difference between the composite feature classifiers and the single genes classifier is the size of the training set. Recall that in the cross-validation procedure we train on one data set and then test on another data set. We repeat this procedure for all possible pairs of data sets; excluding, of course, training and testing on the same data set (*paired setting*). We can, however, also follow an alternative scheme where we train on *all* data sets except the test set, the so-called *merged setting*. More specifically, in this setting four of the five Affymetrix data sets were merged to form a single training data set and the fifth data set was used as test set. Thus, we receive for each feature selection method five AUC values. This increases the size of the training set, and by comparing the results obtained in this setting with the results from the paired setting, we can investigate the effect of the training set size on the classifier performances.

**Figure 9:**
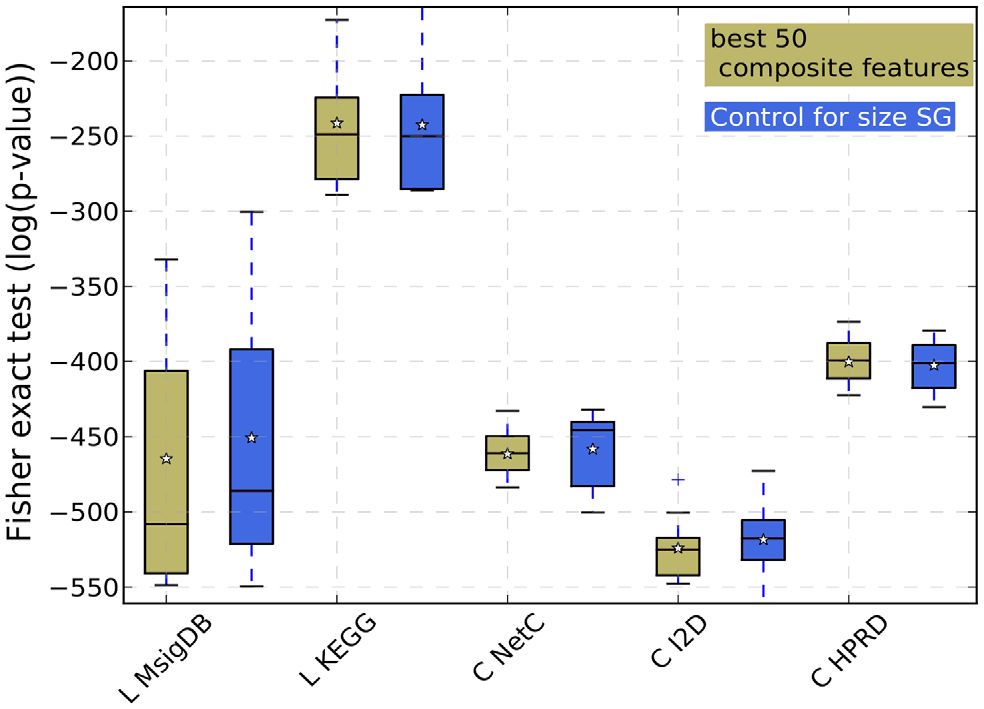
Feature stability when corrected for gene set size. Box plots of the p-values of the Fisher exact test computed for all pairs of gene sets derived from two different data sets. The green box plots represent the values for genes constituting composite features, while the blue box plots (denoted as ‘Control for size SG’) represent the gene-size-corrected values for single genes classifiers. The white stars represent the means of the distributions.

Figure 5 depicts the results for the merged setting and the pairwise setting for the CV-optimized feature sets and when only the top 50 features are selected. (Note that, in contrast to the results in Figure 2, this pairwise setting only employs the Affymetrix datasets). The results for the top 100 and 150 features are similar, see Figure 9 in File S1. Statistical testing shows that in the paired setting (Tables 16–19 in File S1) when the number of features is set to 150, *Lee* employing the MsigDB performs better than the single genes classifier. However, this difference disappears when correcting for multiple testing. More importantly, there are no significant differences between the performances of the single genes and composite feature classifiers in the merged setting (Tables 20–23 in File S1). Hence, we can also reject the hypothesis that the lack of performance difference is due to the sizes of the employed training sets.

#### Dataset homogeneity affects single genes and composite classifiers similarly

Breast cancer is a collection of several heterogeneous diseases that show very different gene expression patterns [14]. Expression patterns predictive of outcome might vary between subtypes, which typically leads to problems when training classifiers on gene expression data derived from breast tumors. If this is not explicitly taken into account during classifier training it could result in poor performance and unstable classification, as the selected genes may depend on the composition of the training set. In this section we control the heterogeneity in both the training and test sets by only selecting the relatively homogeneous ER positive breast cancer sub-population. Since the training sets become too small in the paired setting if we only select the ER positive cases, we followed the merged setting outlined above. More specifically, we created a test set consisting of all ER positive cases of a single data set and a training set by pooling all ER positive cases from the remaining data sets. We repeated this procedure across the six data sets and thus obtained six AUC values per classifier. Figure 6 depicts the results for the CV-optimized feature sets and the top 50 features. As before, the classifiers employing a fixed number of features perform similar to classifiers based on a feature set optimized in the CV procedure. See Figure 6B, and also Figure 10 in File S1. In general, and in accordance with earlier observations as made, *e.g.*, by Popovici *et al.* [10], the performance of all classifiers is substantially better on the ER positive cases compared to the unstratified case. More importantly, also in this setting there are no significant performance differences between the single genes classifiers and composite feature classifiers (Tables 24–27 in File S1).

### Equal Classification Using Real or Randomized Networks and Pathways

In the previous sections we showed that composite classifiers do not perform significantly better than classifiers employing single genes as features. We investigated several factors that could influence the performances of these two approaches, but failed to find any factor that induces significant performance differences on the data sets we employ in this study. This lead us to question whether prior knowledge sources really contain information that is of value in constructing features for classifiers predicting outcome in breast cancer. Chuang *et al.* [3] compared their PPI-based classifier to classifiers derived from randomized PPI networks. They concluded that their classifier performed significantly better than random classifiers. We decided to repeat this analysis for a subset of the classifiers in our comparison to determine whether prior knowledge sources really contain information relevant for predicting outcome in breast cancer. To this end, we generated, for each prior knowledge source, 25 random instances. More specifically, we maintained the structure of the pathways, networks and gene sets, and randomly permuted the identities of the genes. In doing so, the original topology of the secondary data is preserved while the biological information is destroyed. We then repeated the whole validation procedure on all 25 random instances for the feature extraction methods *Lee* and *Chuang*. The results of this analysis are presented in Figure 7. Strikingly, classifiers derived from secondary data sources suffer no significant performance degradation when employing randomized secondary data sources. The performance of *Chuang* on randomized PPI data clearly has a large variance, and there are instances of classifiers derived from random networks that perform much worse and much better than classifiers derived from the non-randomized networks. Furthermore, we found that most classifiers based on randomized secondary data show performances similar to the classifiers derived from the non-randomized secondary sources. To formalize this observation, we performed a statistical test. We have reason to believe that the results derived from the real data should be better than the results derived from random data. Hence we performed one-sided paired Wilcoxon rank tests to determine whether the null hypothesis that the mean ‘non-randomized’ AUC-value is larger than each of the the 25 ‘randomized’ mean AUC-values can be rejected. We performed a Bonferroni correction to account for multiple testing. The results in Figures 11–13 in File S1 and Tables 28–33 in File S1 show that in the vast majority of the cases the null hypothesis can not be rejected. Conversely, it is very simple to generate a randomized secondary data source that performs equally well as the real data source. This result shows that further research must be done on the utility of secondary data sources in predicting breast cancer outcome.

### Current Composite Feature Classifiers do not Increase the Stability of Gene Markers

Apart from performance improvements, it is also frequently claimed that features derived from classifiers employing secondary data sources are far more stable than single genes classifiers. In other words, whereas single genes signatures extracted from different data sets show very little overlap, features extracted by approaches that employ secondary data sources are claimed to show a large degree of overlap, even though the features were derived from separate data sets. We determined whether feature sets extracted from secondary data sources do, in fact, show a larger degree of stability than single genes feature sets. We employed the Fisher exact test as a measure of overlap. We use the Fisher exact test since it has the desirable property of automatically compensating for the size of the population from which the signatures, for which the overlap is being computed, are selected. Here, taking these sizes into account is crucial since the starting gene sets defined on the one hand by the genes present on the microarray (single genes approach) and on the other hand by the secondary data sources (composite features) vary immensely. Please note that in this specific context we do not employ the Fisher exact test to test a certain hypothesis. We rather interpret the p-values returned by the test as a measure of enrichment or overlap between two sets of marker genes. Thus, the smaller the p-value the more unlikely the overlap. In addition to the Fisher exact test, we calculated the overlap between gene marker sets by employing the Jaccard index and compensated for the size of the population from which the signatures were selected by a random sampling procedure. See Section 7.2 in File S1 for more details. We performed the overlap comparison for the cases where the top 50, 100 and 150 features are selected. Figures 8 and 15 in File S1 illustrate the results for the Fisher exact test. To assess whether there exist differences in the distributions, we performed Wilcoxon rank tests (see Table 34 in File S1). Indeed, these results confirm that *Chuang*-NetC, *Chuang*-IPP and *Chuang*-HPRD produce more stable gene signatures across datasets. However, strictly speaking, such a comparison compares the proverbial apples and oranges, since a single composite feature can contain many genes. In order to ensure that the low overlap of single genes is not only due to the fact that the best single gene feature sets contain fewer genes than the other feature sets, we controlled the single gene feature sets for size. More specifically, for each data set and each feature selection approach employing secondary data sources, we obtain a single best feature set consisting of 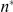 features (networks, gene sets or pathways) where each feature, in turn, consists of *m* genes. We then determine a size-matched single genes set by choosing the best *m* single genes on that same expression data set. The results are shown in Figures 9 and 16 in File S1 for the Fisher exact test and in Figure 17 in File S1 for the approach combining random sampling with the Jaccard index. The Wilcoxon rank test on the Fisher exact results (Tables 35–37 in File S1) shows that only features determined with *Lee* -MsigDB have a higher overlap between datasets than the single genes sets. For the approach combining random sampling with the Jaccard index (Tables 38–40 in File S1) none of the composite feature approaches shows a significantly larger overlap than the single genes approaches. In fact, in four of the 14 experiments single genes approaches show a significantly larger overlap (after correcting for multiple testing) than the *Lee* approach. We can therefore conclude that - when properly size matched - singe genes classifiers produce feature sets that are at least as stable as composite feature classifiers.

## Discussion

In this study we evaluated the prediction performance of network and pathway-based features on six breast cancer data sets. In contrast to previous studies we found that none of the classifiers employing composite features derived from secondary data sources can outperform a simple single genes classifier. Moreover, we did not find any evidence that composite features show a higher stability across the six breast cancer cohorts. Our findings suggest that with the feature extraction methods tested in this study, we cannot extract more knowledge from secondary data sources than we find in the expression of single genes.

There are several issues that could potentially contribute to that situation. First, secondary data sources are, to a large degree, generated by high-throughput biological experiments and could thus contain a level of noise that deems them inappropriate for outcome prediction in breast cancer. On the other hand, the search algorithms could be unsuitable to detect biologically meaningful networks. All three feature extraction methods only extract local information without taking into account the full structure of the network or pathway data. This local information is then combined in the classifiers in a rather crude way, namely by simply averaging the expression of the genes associated with a feature that was found to be associated with outcome, *i.e.* treating each single sub-network or sub-pathway as a single dimension in the classification space. Possible dependencies between features are not taken into account. Also, exploring the subnetwork search space in a heuristic manner may decrease classification performance. The recent method by Dittrich *et al.* [15] computes provably maximally deregulated connected subnetworks based on a sound statistical score definition. This method has not yet been used for classification.

Other recent algorithms as presented by Ulitsky *et al.* [16], Chowdhury *et al.* [17] and Dao *et al.* [18] search for deregulated subnetworks in subsets of samples. These subnetworks are sets of genes that are deregulated in most, but not necessarily all, patients with poor disease outcome. The heuristic method by Chowdhury *et al.* [17] has been shown to perform well on cross-platform classification of colorectal cancer outcome. Dao *et al.* [18] improved on these results by exact enumeration of all dense subnetworks with the above-mentioned property. Looking at subsets of samples in a class, *i.e.* a subset of the poor outcome samples, is an interesting aspect for further evaluation, in particular for breast cancer outcome prediction as it may quite accurately capture the large phenotypic variety of this rather inhomogeneous disease.

Lee *et al.* [4] and Chuang *et al.* [3] average the expression values of single genes comprising a subnetwork, to determine the ‘activity’ of the subnetwork. This is, however, a very simplistic view of the dynamics in a subnetwork itself. In contrast to these two algorithms, the method by Taylor *et al.* [5] predicts outcome by trying to capture the disruption of the regulation of a hub protein over its interactors in poor outcome patients. This is implemented by using every edge leading to a hub as a feature in classification space. Yet, in this way, the classification space becomes too large to allow for good classification results. This method is thus not appropriate for solving the classification problem and this is clearly demonstrated by the poor performance of this algorithm in the comparison.

To find a subnetwork scoring function remains one of the biggest problems when including promising gene sets into a classification framework. Abraham *et al.* [7] tested the classification performance of gene sets provided by the MsigDB. The authors employed several set statistics like mean, median and first principle component to score the gene sets. They found that none of the classifiers employing gene sets and scoring them with the set statistics performed better than a single genes classifier.

In our experiments where we shuffle the genes in the secondary data we showed that features determined on this nonsensical biological data perform equally well in classification than features determined on the real secondary data. This again could possibly be caused by the low quality of the network and pathway data. However, the nature of gene expression patterns in breast cancer, and specifically its association with outcome can also explain these findings. Since many genes are involved in breast cancer and are differentially expressed and associated with outcome, as shown, for example by Ein-Dor *et al.* [19], the chance that those genes span decently sized subnetworks in the randomized secondary data is high. Both algorithms, *Chuang* and *Lee*, look for highly differentially expressed subnetwork or pathway markers, and these can also be found in the randomized data. Furthermore, overlaying networks or pathways that contain protein level information with mRNA expression data might result in erroneous results. These data sources reflect events on very different molecular levels. While gene expression and protein expression is undoubtedly linked, there are many processes that prevent this from being a trivial one-to-one mapping. Thus, we may measure, for a set of genes, an effect on the mRNA level that leads to differential expression between the two patient classes but this may have little bearing on the relationships between these genes as captured in the PPI graph. In conclusion, our results show that it may not be sufficient to search for sub-networks or sub-pathways that are differentially expressed *on average* but that complex interactions between entities as well as the more complicated relationships between mRNA levels and the topology of the PPI graph need to be taken into account.

Our different classification results are partially owed to the fact that we used a different cross-validation procedure, which, in our opinion, fits the clinical situation, better. The studies by Chuang *et al.* [3] and Lee *et al.* [4] also determined possible features on one data set. However, in contrast to our work they reranked the features on the second (test) data set. Furthermore, they determined the number of features and the classification performance on this second data set using five-fold cross-validation. In our opinion, this does not represent a *bona fide* independent validation of the classifier as the training and test sets are not strictly separated. An advantage of the approach employed by Chuang *et al.* [3] and Lee *et al.* is that differences between platforms can more easily be accommodated. This is less of a problem in our study as five of the employed datasets in our study come from the same Affymetrix platform (U133A) and only one dataset from the Agilent platform. Figure 5 (pairwise setting) and Figure 2 show that the influence of the single Agilent dataset on the classification result is only mild and affects all of the feature extraction methods in a similar fashion.

Also the usage of various types of classifiers in this study contributes to the different findings. Here we tested the NMC, LOG and a 3NN classifiers. While the LOG classifier generally performed much worse than the NMC, it did yield better classification results when employing features extracted with *Lee*. In general, this hints at the fact that the choice of classifier influences the ranking of the feature extraction methods. However, the NMC and 3NN achieved similar performances for all feature extraction methods. Moreover, in accordance with Popovici *et al.* [10], we found that a simple NMC performed best, independent from the feature extraction method.

In summary, we introduced a framework to test the use of feature extraction methods with respect to the prediction of their determined features. We used this framework to specifically test the superiority of feature extraction methods based on network and pathway data over classifiers employing single genes. Across six breast cancer cohorts, we showed that the three tested methods do not outperform the single genes classifier nor do they provide more stable gene signatures for breast cancer.

An important aspect that hampers progress in the field of network and pathway based classification is the lack of proper evaluation of proposed algorithms. In our opinion this is caused by (1) lack of reproducibility of the results; (2) lack of large and standardized benchmark sets to test proposed algorithms and (3) lack of a standardized, unbiased protocol to assess the performances of proposed methods on the benchmark sets. To overcome these issues, we have created a software pipeline that implements all the classifiers as faithfully as possible and also runs our validation protocol. We have also established a large collection of breast cancer datasets (and this is currently being expanded) on which the algorithms can be tested. Both the datasets and the pipeline are freely available. In the long term, we envision a web service where a classifier can be submitted as a software package. The server will then autonomously evaluate the performance of the classifier using the standardized pipeline on the benchmark set.

## Materials and Methods

### Microarray Data

The microarray data sets used in this work is listed in Table 2. To combine the five Affymetrix arrays with the Agilent arrays we first matched the probes on the arrays to Entrez GeneIDs. Only those genes were included in the feature sets that appeared on both platforms, resulting in 11601 genes in total. In case that several probes on one chip matched to the same gene the expression values of the probe with the highest variance was taken. The final expression matrices were then z-normalized such that the expression distribution of each gene has a mean of zero and a standard deviation of one. Samples in the data sets were labeled ‘good’ outcome if no event, that is, a distant metastasis or death, occurred within five years. Otherwise samples were labeled ‘poor’ outcome.

**Table 2:** Microarray expression data.

Dataset	Outcome	good/poor	platform
Chin [26]	Metastasis within 5 years	68/29	Affymetrix
Desmedt [27]	Metastasis within 5 years	91/29	Affymetrix
Loi [28]	Metastasis within 5 years	92/28	Affymetrix
Miller [29]	Death within 5 years	156/37	Affymetrix
Pawitan [30]	Death within 5 years	120/22	Affymetrix
Vijver [13]	Metastasis within 5 years	178/70	Agilent

Expression data used in this study. All data sets were processed as described in [20] and contained 11601 genes with z-normalized expression values afterwards. Column ‘poor/good’ contains the number of samples with poor or good outcome, respectively.

We found that probes matching several geneIDs in the Affymetrix datasets were not discarded, instead the smallest geneID was used to map the probe to a certain gene. This could potentially influence the mapping from gene expression to the network or pathway data and thus, influence the classification result and overlap of gene markers. However, classification on the corrected expression data, *i.e.*, removing these ambiguous probes from the expression matrices and re-executing our cross validation procedure for all of the methods, yielded the same performances and overlap between markers. ER status of patients was predicted from the expression values of the gene ESR1. For more detail of the processing of the data see van Vliet *et al.* [20]. In Section ‘Dataset homogeneity affects single genes and composite classifiers similarly’ we merge datasets to obtain a larger training dataset. This merging was done by pooling all patients from all but one dataset, followed by a z-normalization of the merged datasets.

### Network and Pathway Data

All feature extraction methods were run on the databases KEGG [21] and HPRD [22]. The algorithm by Lee *et al.* [4] was also run on the MsigDB C2 database [9].

#### KEGG

We collected all pathway information available for *Homo sapiens* (hsa) from the KEGG database, version December 2010. The entries contained information on metabolic pathways, pathways involved in genetic information processing, signal transduction in environmental information processing, cellular processes and pathways active in human disease and drug development. We obtained 215 pathways. The nodes contained in the pathways were matched with the KEGG gene database such that each node carries an Entrez GeneID. In this way we obtained a network composed of 200 pathways containing 4066 nodes and 29972 interactions of which 3110 nodes are also contained in the expression sets.

#### MsigDB

As second pathway database we used the C2 collection of the MsigDB (version 3) [9], which was also used in Lee *et al.* [4] (version 1.0). It contains gene sets from online pathway databases such as KEGG, gene sets made available in scientific publications and expert knowledge. We obtained 3272 gene sets of which 2714 could be entirely or partially mapped the six data sets. The MsigDB does not contain any edges, thus this database was only usable for the algorithm by Lee *et al.* [4].

#### HPRD

The HPRD (version 9) provides information on protein-protein interactions (PPI) derived from the literature. The HPRD contains 9231 proteins and 35853 interactions. The proteins were mapped to their genes carrying Entrez GeneIDs. There are 7390 genes contained in both the HPRD and the expression sets.

#### OPHID/I2D

The OPHID/I2D database, downloaded in April 2011, contains protein-protein interactions derived from BIND, HPRD and MINT as well as predicted interactions from yeast, mouse and *C. elegans*. The database contains 12643 nodes and 142309 edges. 9453 of the nodes are also present in the six breast cancer studies examined here.

#### Protein-protein interaction network by Chuang *et al.* [3] (NetC)

The network curated by Chuang *et al.* [3] consists of 57228 interactions and 11203 nodes of which 8572 are contained in the six breast cancer studies. The network is curated from yeast two hybrid experiments and interactions predicted from co-citation.

### Algorithms

#### Notation

Let 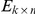 be a gene expression matrix, as we obtain it from a microarray study, with *k* samples and *n* genes. Each entry 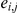 contains the expression value of gene *j* in sample *i*. All samples carry a binary class label 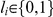 denoting outcome, where 1 denotes ‘poor outcome’ and 0 denotes good outcome'. The label vector of all samples is denoted by 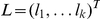. We denote a network by 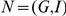 where *G* is the set of genes in the network and *I* is the set of interactions between these genes, also called edges in the following. We define a subnetwork as the connected graph 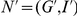 with 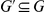 and 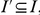 and a pathway as a gene set 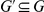. Let 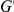 be such a pathway or the set of genes in a subnetwork then according to [3], [4] the activity of the pathway or subnetwork in sample *i* is given as
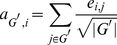
(1)


#### Feature extraction method by Chuang *et al.* [3]

Given a network 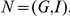 the algorithm by Chuang *et al.* [3] carries out a greedy search starting from a seed–a single gene in the network. It then iteratively extends the network by adding neighboring genes to find subnetworks with high mutual information (MI) of the activity of the pathway and sample labels. Each node in *N* is used once as seed. In each step, an additional gene is picked that leads to a maximal MI improvement. If no improvement is possible, the search stops.

More precisely, the association between the subnetwork activity and the class labels is computed as follows: Given a subnetwork 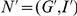 the activity vector 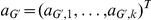 is calculated using Equation (1). To compute the MI, vector 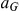 is discretized. Given a dissection of the interval 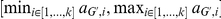 into 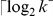 bins let 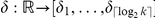 be a function that assigns a network activity to a sample with one of these bins, where 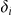, 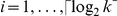, denote the bins. We define the mutual information 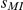 between the probability density of the bins 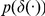 and the probability distribution of the class labels 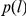 as
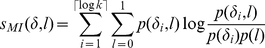
(2)where 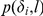 is the joint distribution of 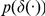 and 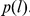 The algorithm also performs three statistical tests to extract only networks that show significantly high mutual information. The ranking of the networks is given by ordering the networks according to their mutual information 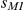.

In our study we use PinnacleZ, an implementation of the algorithm provided by the authors. As feature values for classification the subnetwork activity as given in Equation (1) of the determined subnetworks was used.

Before determining the subnetworks, PinnacleZ performs a z-normalization of the given data set. This is undesirable when looking at subsets of data sets as we do in the five-fold cross-validation. In order to skip the normalization step, we implemented a patch in the PinnacleZ source code. This patch adds a “-z” option that instructs PinnacleZ to *omit* its usual gene-wise z-normalization step.

One problem when mapping the expression data to the network data is that for some nodes there is no expression data. Chuang *et al.* [3] do not state in their paper how they handled this problem although their identified subnetworks contain such nodes. We therefore filtered out proteins for which no expression data is available before running PinnacleZ. For further issues we encountered when working with PinnacleZ see File S1.

#### Algorithm by Lee *et al.* [4]

The algorithm by Lee *et al.* [4] uses the t-statistic to rank pathways according to their overall differential expression. For this it first defines sets of genes, the so called condition responsive genes (CORGs), which contain the most differentially expressed genes of a pathway. These genes are found by applying a greedy search. For each pathway the genes are ordered according to their t-statistics. Given the two sample groups let 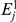 be the expression values of gene *j* for all samples with class label 1 and 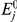 the expression values of gene *j* for all samples with class label 0, respectively. Let 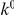 and 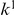 denote the number of samples in each group; 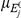 and 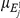 denote the means of the two groups and 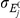 and 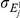 the standard deviation in the two groups. The t-statistics between 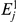 and 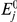 is given by
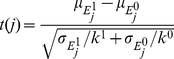
(3)


The genes in a pathway are sorted either in ascending order, if the highest absolute *t* value is negative, or in descending order, if the highest absolute *t* value is positive. Given this order the greedy search iteratively combines genes and calculates their average expression, or *pathway activity*, across the samples as it is given in Equation (1), *i.e.* 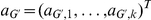 is calculated where 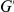 contains the best *m* genes according to the ranking. To evaluate the combined discriminative power of the genes that have been averaged, the t-statistic of the averaged expression is computed as follows:
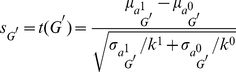
(4)where 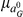 and 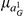 represent the means and 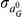 and 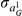 represent the standard deviations of the averaged activities. If the resulting value 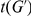 is higher than the previous value of the t-statistics then the search continues adding the gene to the already determined CORGs, otherwise the search stops. The score 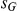 of the final CORGs 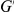 is then used to rank the pathway.

As mentioned beforehand, *Lee* can only be executed on predefined gene sets. Those gene sets are normally not provided in a PPI database. Thus, we used the KEGG and MsigDB databases to evaluate this algorithm. In order to decrease the running time the authors executed a pathway ranking by employing the algorithm by Tian *et al.* [23] and just taking the top 10% of pathways into account prior to executing their algorithm. In our setting we ranked all of the pathways according to the algorithm by Lee *et al.* [4] and considered for determining the optimized number of features in the final classifier all pathways in KEGG and the top 400 pathways in MsigDB. As feature values for classification the pathway activity, as computed according to Equation (1) for all condition-responsive genes (CORGs), is employed. Here again we excluded proteins in the pathways for which no expression data is available.

#### Algorithm by Taylor *et al.* [5]

In contrast to the algorithms by Chuang *et al.* [3] and Lee *et al.* [4], the algorithm by Taylor et al. [5] first identifies organizer proteins in the network, the so-called hubs, and then attaches a weight to each edge between the hubs and their direct neighbors in the network. These weights are later used to train a classifier.

Candidate hubs are the 15% most densely connected proteins in the network data, independent from their expression status. For the following calculations proteins without expression data are excluded. To identify hubs that significantly change their interaction behaviour between the two classes the authors introduce the hub difference and the average hub difference which are based on the Pearson correlation. The Pearson correlation between a hub *h* and an interactor *n* of this hub is defined as the Pearson correlation between their expression profiles across the *k* samples
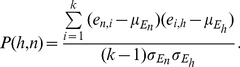
(5)



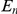
 and 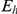 denote the distribution of expression values across the *k* samples and 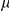 and 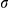 are their means and standard deviations. The hub difference is defined as the difference of the Pearson correlation 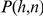 given the two sample classes, indicated by the superscript 0 and 1,
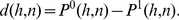
(6)


Let 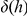 denote the set of neighbors of a hub *h* then the average hub difference is
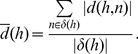
(7)


To extract only those hubs that show a significant average hub difference the value is compared to a distribution of the average hub difference for a permuted dataset, using a p-value cut off of 0.05. This distribution is calculated by 1) randomly permuting the class labels and 2) recalculating the average hub difference and repeating these two steps 1000 times. The significant hubs are ranked by their average hub difference.

As feature values in the classifier differences of the expression of the hub and each of its interactors were used. For example, suppose 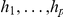 were found significant and suppose.


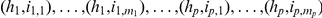
 are the edges to their interactors. Then for one sample the vector 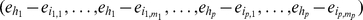 contains the feature values for the classifier.

Since the edges attached to a hub are not ranked, all those edges were included in the classifier, given that the hub shows a significant average hub difference. For the cross-validation procedure this means that we can not train the number of features but only a number of feature sets.

#### Classifiers

In our study we employed a nearest mean classifier (NMC), logistic regression (LOG) and a 3-nearest neighbor classifier (3NN). As distance metric for the NMC we employed the Euclidean distance. More specifically, a sample is projected on the line connecting the two class means, and the Euclidean distance of the projected sample to each class mean is computed. The sample is assigned to the closest class. In addition to the NMC we executed all features extraction methods in combination with the LOG. We found that simple LOG without any regularization parameters cannot be trained properly since for higher numbers of features (approximately 50 features and more) the training step does not converge on the breast cancer data. Moreover, we found that for many features different imfplementations of LOG return different weight vectors. Thus, we used three different implementations (the R GLM, R NNET and Python SciKits implementation) and only accepted the classification result of the R GLM implementation when all three versions converged to the same weight vector. Furthermore, we tested the classification performance of pathway and network based features and single genes in a 3-nearest neighbor classifier (3NN). As distance metric we chose the Euclidean distance. Further, we weighted the contribution *w* of each neighbor to a sample’s score by
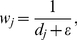
(8)where 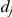 denotes the Euclidean distance of the *j*th neighbor and 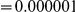. The results of this classifier are presented in the Supplement Section 1.2.

#### Cross-validation and classification

In the cross-validation procedure we employed, we rigorously separate the training and test data sets. For details, see Figure 1 and Algorithm 1.

#### Algorithm 1

Cross-validation procedure.

**Require: Datasets** 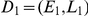, 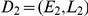 where 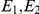 are expression matrices and 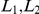 are vectors of outcome labels.

**Feature extraction method** 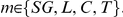


**Secondary data source** *N*.

**Classifier** 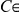 {logistic regression, nearest mean classifier, 3-nearest neighbour classifier}.

/* Cross-validation on 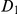 */.

1: 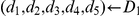 split samples in 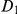 into 5 parts 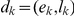 where 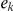 denotes the expression values of the samples in split *k* and 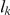 their class labels */
2: **for** 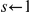 to 5 **do**
3: 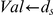/*validation set 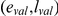 */
4: 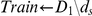 training set 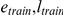 */
5: 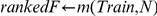 determine features according to the published methods and rank them according to the appropriate score or determine the ranking of the single genes according to the t-statistic on *Train* */
6: 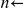 number of *rankedF*
7: 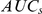 = empty list
8: **for
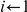
** to *n* **do**
9: 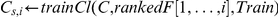 train the classifier with *i* features */
10: 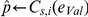 ranking of the samples in the validation set */
11: 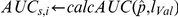 calculate AUC value for 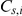 */
12: **end for**
13: **end for**

/* train CV-optimized classifier on 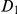 and validate it on an independent data set 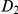 */.

14: **for 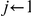
** to *n* **do**
15: 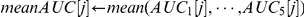

16: **end for**
17: 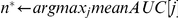

18: 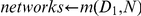

19: 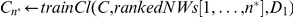

20: 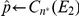

21: 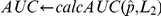

22: **return** *AUC*

The training phase consists of determining the best performing number of features and training the final classifier with this number of features. The trained classifier is then tested in the test phase. The data sets used in these two phases are completely independent, *i.e.* the test set is never used in training the classifier.

To determine the optimized number of features in our classifiers we employed five fold cross-validation. In this cross-validation, we first determined all required composite features (if necessary) and their ranking on four splits of the training data set. Then a series of classifiers is trained on the same four splits by gradually adding features according to the ranking. These classifiers are then evaluated on the fifth split of the data set. Since this is done in a five fold cross-validation we obtain for each of the classifiers five different AUC values. The optimized number of features extracted corresponds to the number of features yielding the highest mean performance. Once the best performing number of features is determined, the features are calculated using the whole training data set and the final classifier is trained also using the complete training data set. The classifier is then tested on the test data set. For each method we used all possible pairs of data sets as training and test set respectively. Since we have six data sets available this resulted in 30 AUC values for each method.

## Supporting Information

File S1 **Supporting Information File.** The File contains all supporting Figures, Tables and Texts.
(PDF)
