# Supplementary material for: A Critical Evaluation of Network and Pathway-Based Classifiers for Outcome Prediction in Breast Cancer
Source: PLoS One. 2012 Apr 27;7(4):e34796. doi: 10.1371/journal.pone.0034796 (PMC3338754; doi:10.1371/journal.pone.0034796)
Supplement: File S1 — Supporting Information File. The File contains all supporting Figures, Tables and Texts. (PDF) [file pone.0034796.s001.pdf]

Supplement A critical evaluation of network and pathway-based classifiers for outcome prediction in breast cancer

Staiger *et al.*

1 Current composite feature classifiers do not outperform single gene classifiers on six breast cancer datasets

We performed a paired Wilcoxon rank test between the AUC distribution of the single-gene classifier and all composite feature classifiers. Table S1 contains the two-sided p-values of the statistic.

Table S1. P-values of the Wilcoxon rank test. CV-opt features

	C HPRD	C I2D	C NetC	C KEGG	L KEGG	L MsigDB
SG	0.2054	0.0076	0.1840	0.0467	0.9426	0.7953

	T HPRD	T I2D	T NetC	T KEGG
SG	0.0000	0.0000	0.0000	0.0002

Table S2. Number of features used in the cv-opt. NMCs. In parenthesis the number of genes contained in the cv-optimized number of features are given.

	Chin	Desmedt	Loi	Miller	Pawitan	Vijver	mean	std
C HPRD	31(190)	20(118)	282(982)	194(779)	261(840)	26(151)	135.67 (510.0)	113.2 (362.63)
C I2D	315(1319)	396(1500)	10(94)	5(48)	373(1221)	346(1035)	240.83 (869.5)	166.84 (581.20)
C NetC	291(1077)	225(900)	310(1112)	135(624)	230(796)	109(490)	216.67 (833.17)	73.88 (225.30)
C KEGG	63(290)	84(358)	133(356)	7(43)	89(251)	30(130)	67.67 (238.0)	41.05 (116.11)
L KEGG	210(494)	209(489)	213(492)	208(443)	32(103)	203(486)	179.17 (417.83)	65.88 (141.87)
L MsigDB	12(93)	10(60)	11(68)	188(361)	340(516)	371(627)	155.33 (287.5)	155.02 (227.54)
SG	27	6	58	250	147	72	93.33 (93.33)	82.82 (82.82)
T HPRD	87(1473)	98(1659)	17(307)	37(696)	98(1534)	2(32)	56.5 (950.17)	39.334 (637.44)
T I2D	26(1065)	25(1015)	28(1440)	18(1107)	2(82)	20(1178)	19.83 (981.17)	8.69 (424.61)
T NetC	20(551)	5(126)	22(535)	37(781)	31(698)	12(187)	21.17 (479.67)	10.76 (244.05)
T KEGG	35(598)	27(628)	31(764)	42(868)	30(825)	44(539)	34.83 (703.67)	6.26 (122.05)

1.1 Logistic regression

A B

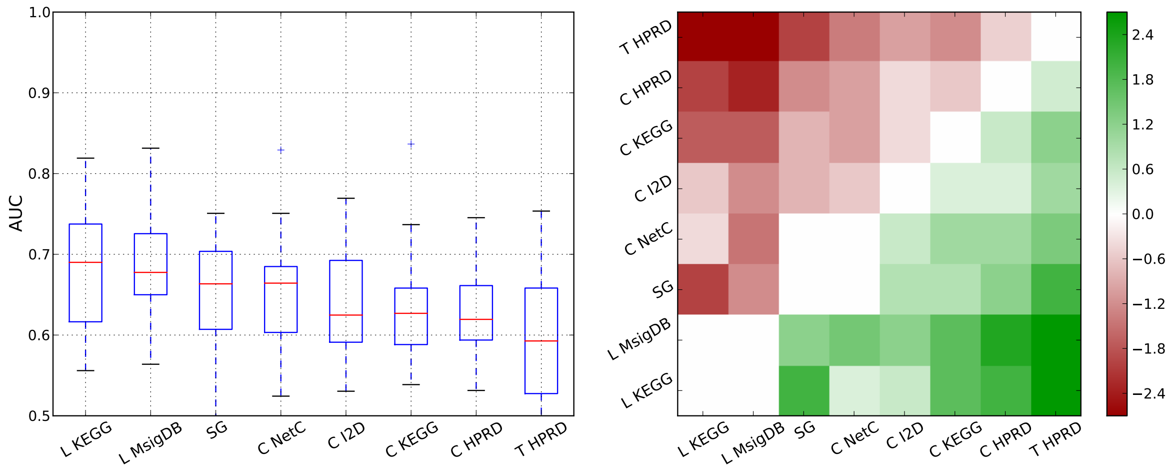

Figure S1. Performances of the LOG classifiers employing single genes and composite features constructed from different secondary data sources.
A: Box plots of the 30 AUC values. The boxes are sorted in descending order according to the median.
B: This panel shows the result of pairwise comparisons between all combinations of feature extraction methods and secondary data sources. If, for a given combination of training and test data set, the AUC value of classifier i is higher (lower) than the AUC value of classifier j on the same test data set, it is counted as a win (loss) for classifier i. Element (i, j) in the matrix represents the $\log_2$ ratio of wins to losses of method i compared to method j. Green indicates an overall win, red an overall loss and white represents draws. The rows and columns are sorted as in Panel A.

Table S3. P-values of the Wilcoxon rank test. LOG classifiers with CV-opt features

	C HPRD	C I2D	C NetC	C KEGG	L KEGG	L MsigDB	T HPRD
SG	0.0248	0.3387	0.8078	0.0841	0.0093	0.0058	0.0006

Since the LOG only performs stably when using few features, an analysis of the performance of the 50, 100 and 150 best features was not possible.

Table S4. Number of features used in the cv-opt. LOG. In parenthesis the number of genes contained in the cv-optimized number of features are given.

	Chin	Desmedt	Loi	Miller	Pawitan	Vijver	mean	std
C HPRD	10(73)	15(92)	10(85)	1(9)	13(96)	48(256)	16.17 (101.83)	14.89 (74.87)
C I2D	6(43)	1(13)	8(76)	12(107)	9(73)	48(290)	14.0 (100.33)	15.57 (89.7)
C NetC	11(89)	17(124)	12(102)	4(31)	12(95)	48(270)	17.33 (118.5)	14.23 (73.41)
C KEGG	16(89)	18(123)	17(91)	24(129)	15(71)	2(15)	15.33 (86.33)	6.62 (37.68)
L KEGG	14(72)	16(101)	6(34)	7(30)	12(39)	2(13)	9.5 (48.17)	4.89 (29.47)
L MsigDB	11(84)	9(47)	4(35)	3(11)	14(46)	4(29)	7.5 (42.0)	4.11 (22.3)
SG	6	6	22	44	23	2	17.17 (17.17)	14.47 (14.48)

1.2 3-Nearest neighbor classifier

In addition to the logistic regression and the NMC we tested the classification performance of pathway and network based features and single genes in a 3-Nearest neighbor classifier (3NN). As distance metric we chose the Euclidean distance. Further, we weighted the contribution w of each neighbor to a sample's score by

$$w_j = \frac{1}{d_j + \epsilon} \tag{1}$$

where d_j denotes the Euclidean distance of the jth neighbor and $\epsilon = 0.000001$.

#features	Boxplot	Winchart
50	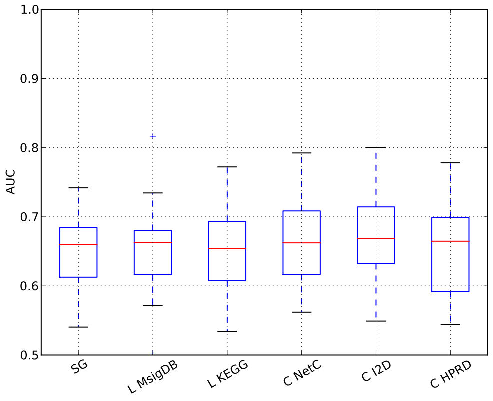	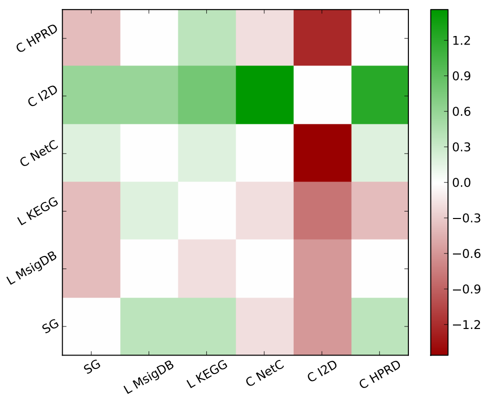
100	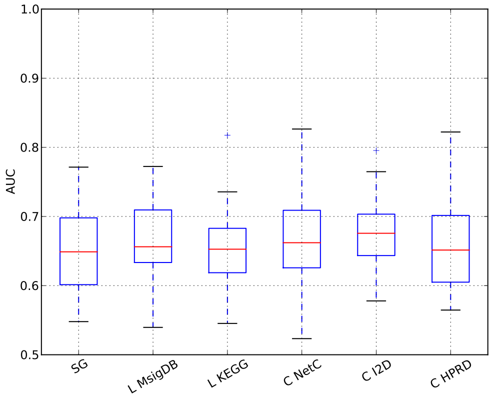	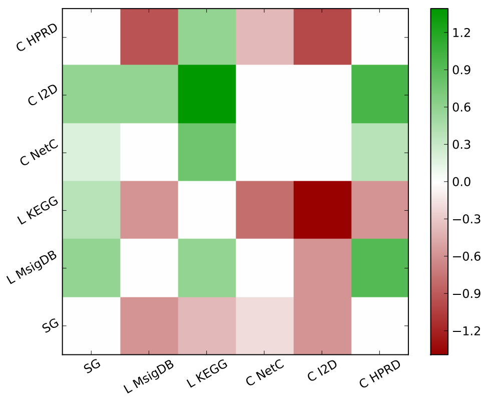
150	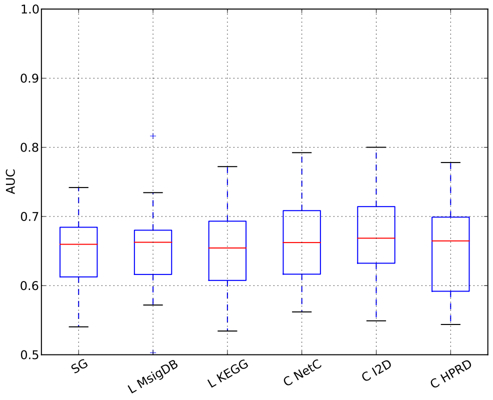	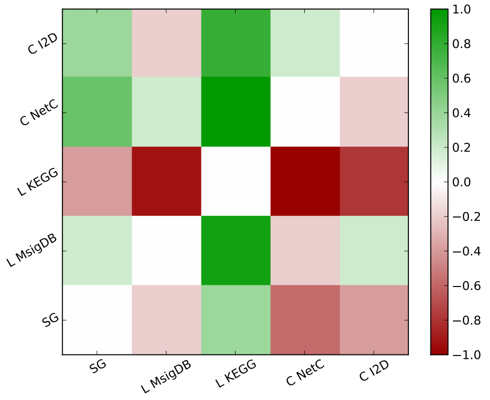

Figure S2. Performances of the 3NN classifiers employing single genes and composite features constructed from different secondary data sources.
First column: Box plots of the 30 AUC values. **B:** This panel shows the result of pairwise comparisons between all combinations of feature extraction methods and secondary data sources. If, for a given combination of training and test data set, the AUC value of classifier i is higher (lower) than the AUC value of classifier j on the same test data set, it is counted as a win (loss) for classifier i. Element (i, j) in the matrix represents the $\log_2$ ratio of wins to losses of method i compared to method j. Green indicates an overall win, red an overall loss and white represents draws. The rows and columns are sorted as in Panel A.

Table S5. P-values of the Wilcoxon rank test. Single genes classifier performances versus all network and pathway based classifier performances.

50 best features						
	L MsigDB	L KEGG	C NetC	C I2D	C HPRD	
SG	0.9838	0.7303	0.5561	0.2054	0.7734	
100 best features						
	L MsigDB	L KEGG	C NetC	C I2D	C HPRD	
SG	0.2534	0.7611	0.3492	0.0919	0.7000	
150 best features						
	L MsigDB	L KEGG	C NetC	C I2D	C HPRD	
SG	0.1840	0.6808	0.1840	0.1142		

1.2.1 Cross validation results: mean AUC vs. number of features

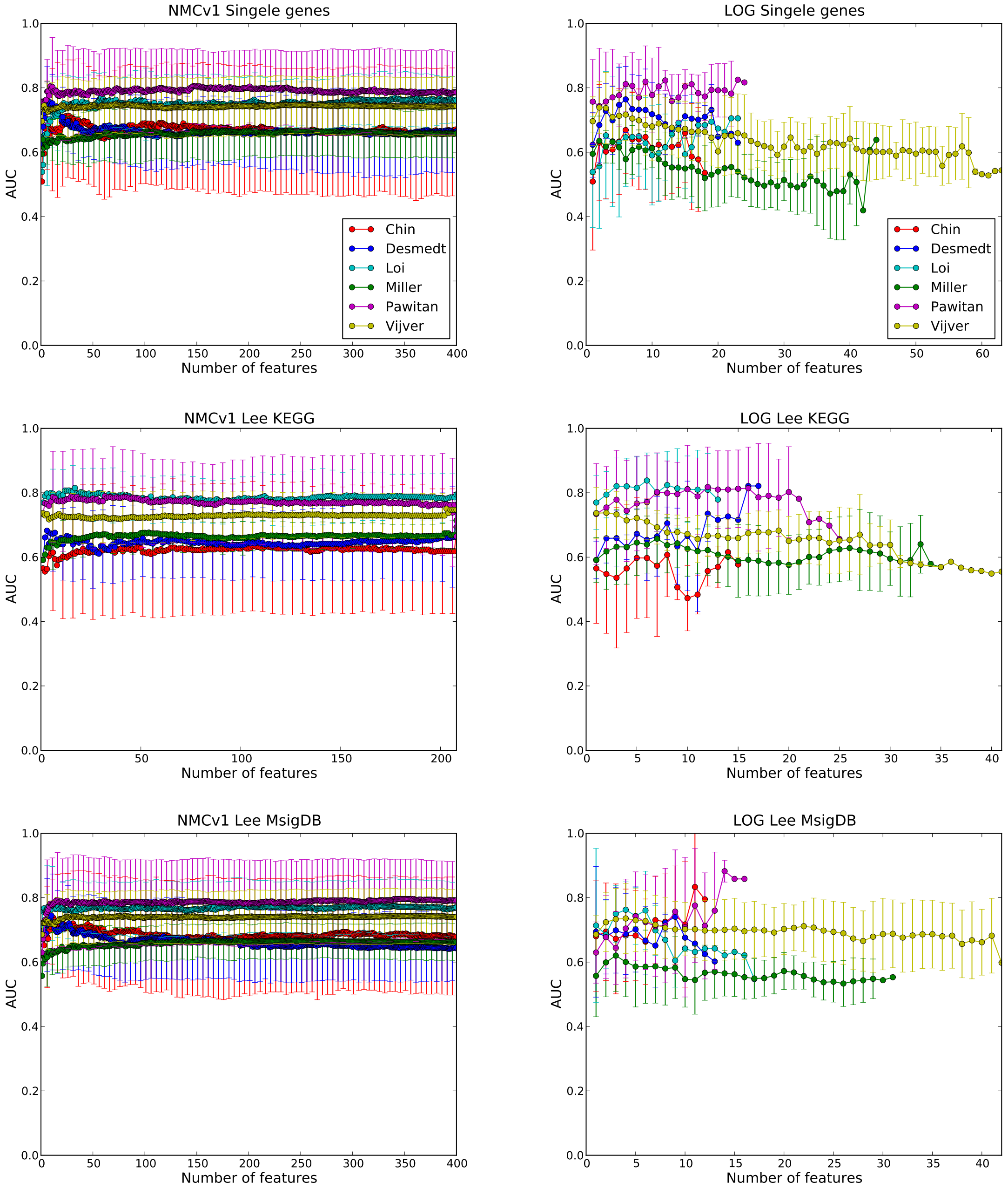

Figure S3. Mean performances from the five-fold cross-validation. For a range of features we calculated the five-fold cross-validation per dataset. Shown are the results from the NMC and the LOG in combination with the single-gene approach and the algorithm by Lee *et al.*

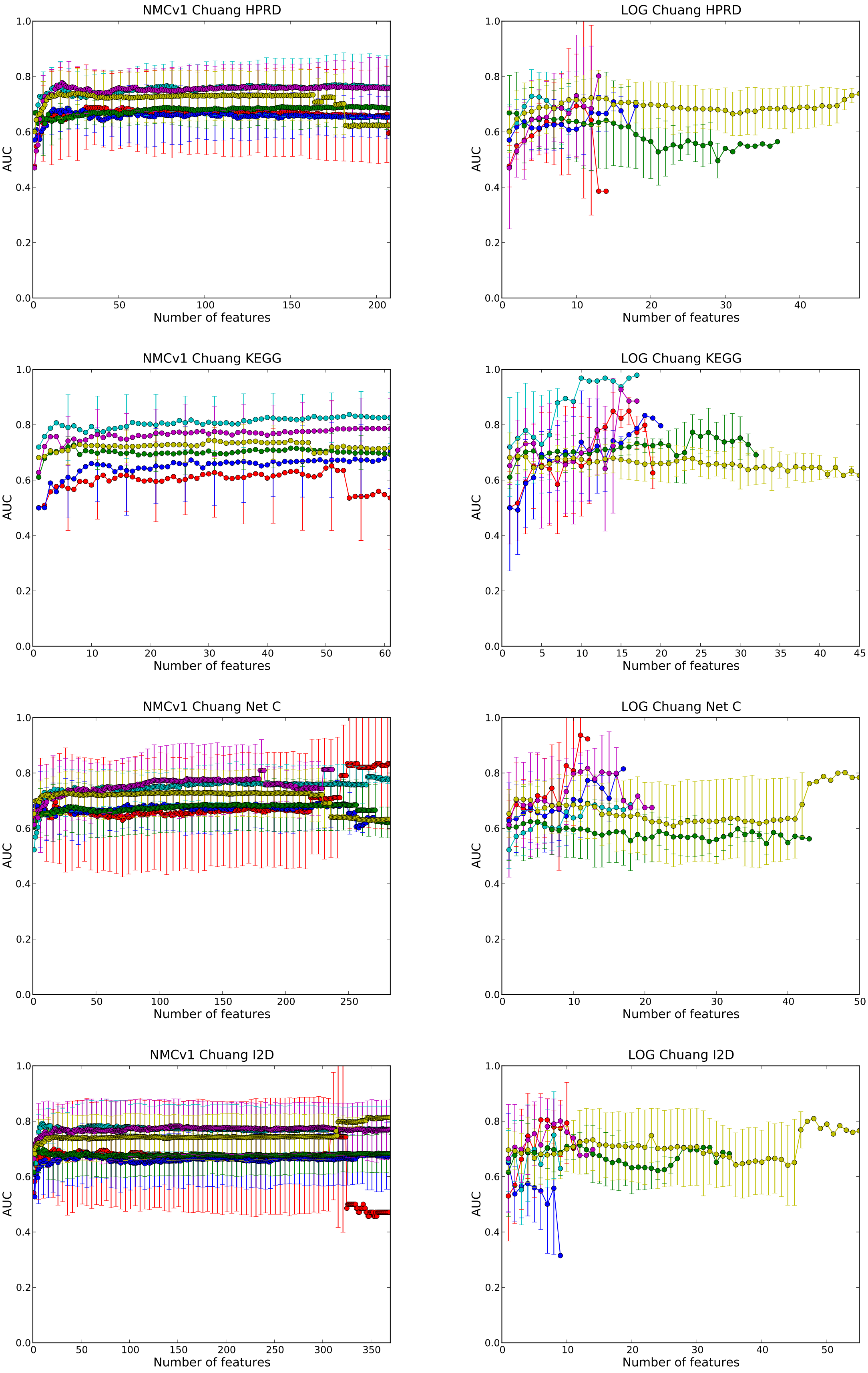

Figure S4. Mean performances from the five-fold cross-validation per dataset. For a range of features we calculated the five-fold cross-validation performance per dataset - one curve per dataset. Shown are the results from the NMC and the LOG in combination with the algorithm by Chuang *et al.* *Chuang* returns, for each cross validation fold, a specific number of features - this may vary across folds. The indicated averages are computed only across the number of folds that returned a value.

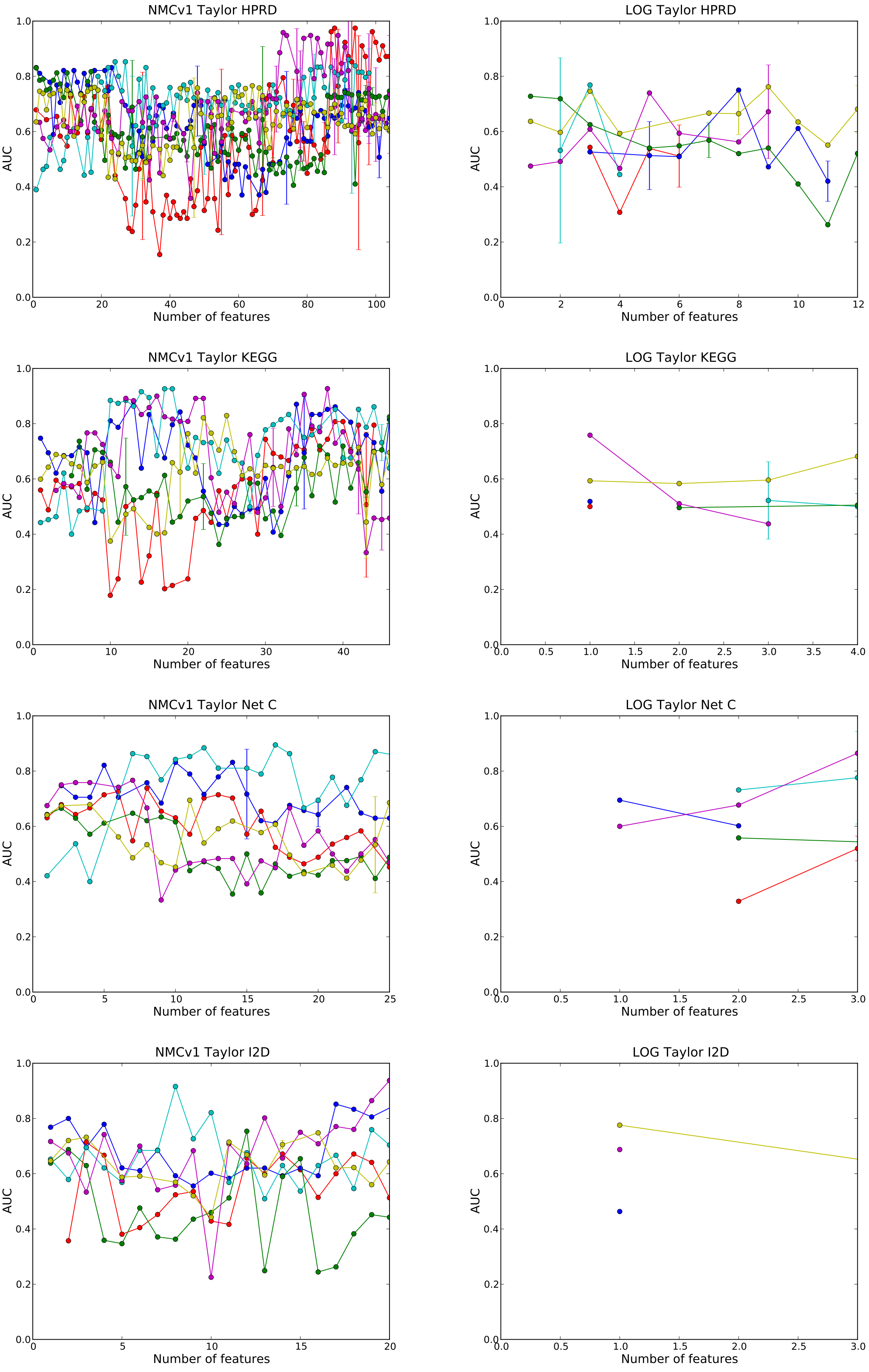

Figure S5. Mean performances from the five-fold cross-validation per dataset. For a range of features we calculated the five-fold cross-validation performance per dataset - one curve per dataset. Shown are the results from the NMC and the LOG in combination with the algorithm by Taylor *et al.* *Taylor* returns, for each cross validation fold, a specific number of features - this may vary across folds. The indicated averages are computed only across the number of folds that returned a value.

2 The number of selected features does not effect the relative performances

Table S6. P-values of the Wilcoxon rank test for the 50 best features. Testing whether mean single gene performance is different from mean composite feature classifier performance.

	L MsigDB	L KEGG	C NetC	C I2D	C HPRD
SG	0.3709	0.7655	0.0405	0.0010	0.0345

We performed a paired Wilcoxon rank test between the AUC distribution of the classifiers with CV-optimized number of features and and their counter parts using the best 50, 100 and 150 features (see Table S7).

Table S7. P-values of the Wilcoxon rank test for the 50 best features vs. CV-optimized features

SG	L MsigDB	L KEGG	C NetC	C I2D	C HPRD
0.1204	0.5440	0.4331	0.0636	0.3759	0.0634

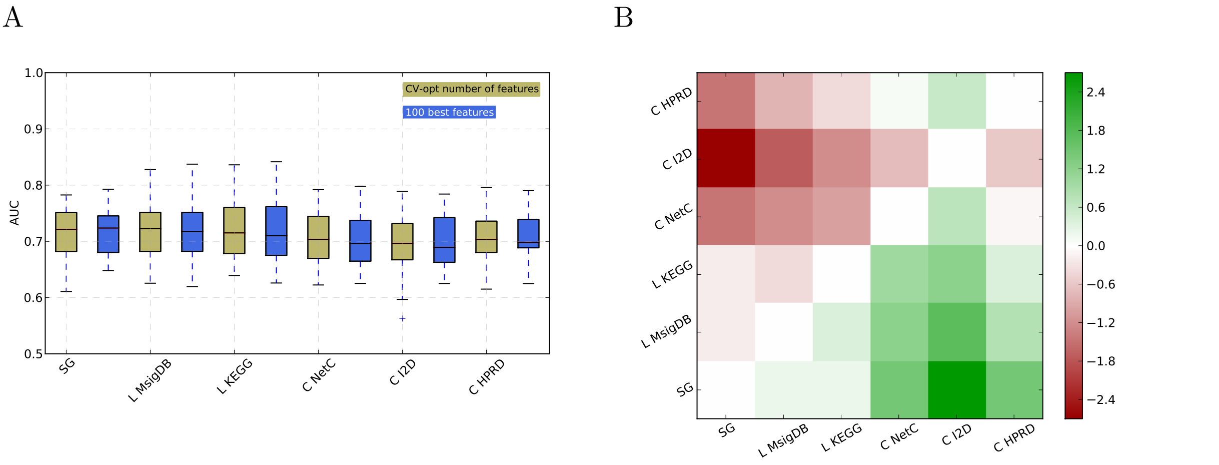

Figure S6. Performances of the NMC classifiers for single genes and composite feature classifiers using 100 features. For each combination of feature extraction method and secondary data source, and each pair of datasets we obtained one AUC value resulting in 30 AUC values per combination. **A:** Each box plot shows the median, the 25% and 75% percentiles and the standard deviation of the 30 AUC values. Outliers are depicted by crosses. The performances associated with the CV-optimized features (100 best features) are depicted by the green (blue) boxplots, respectively. **B:** This panel shows the result of pairwise comparisons between all feature extraction - prior knowledge source combinations. The rows and columns are sorted as in Panel A.

Table S8. P-values of the Wilcoxon rank test for the 100 best features. Testing whether mean single gene performance is different from mean composite feature classifier performance.

	L MsigDB	L KEGG	C NetC	C I2D	C HPRD
SG	0.7303	0.2894	0.0011	0.0001	0.0175

Table S9. P-values of the Wilcoxon rank test for the 100 best features vs. CV-optimized features

SG	L MsigDB	L KEGG	C NetC	C I2D	C HPRD
0.8454	0.1444	0.2685	0.1631	0.5170	0.7036

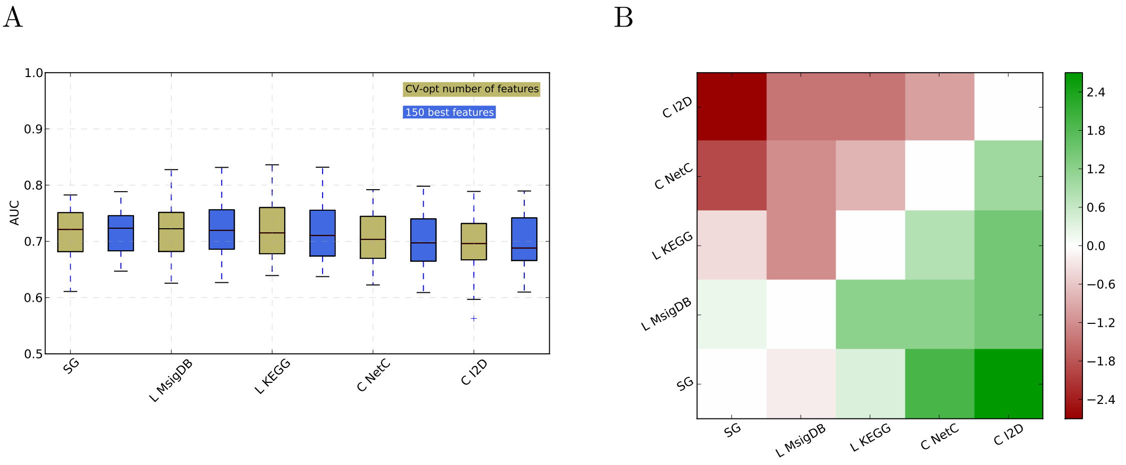

Figure S7. Performances of the NMC classifiers for single genes and composite feature classifiers using 150 features. For each feature extraction - secondary data source combination, and each pair of datasets we obtained one AUC value resulting in 30 AUC values per combination. **A:** Each box plot shows the median, the 25% and 75% percentiles and the standard deviation of the 30 AUC values. Outliers are depicted by crosses. The performances associated with the CV-optimized features (100 best features) are depicted by the green (blue) boxplots, respectively. **B:** This panel shows the result of pairwise comparisons between all feature extraction - prior knowledge source combinations. The rows and columns are sorted as in Panel A.

Table S10. P-values of the Wilcoxon rank test for the 150 best features. Testing whether mean single gene performance is different from mean composite feature classifier performance.

	L MsigDB	L KEGG	C NetC	C I2D
SG	0.2367	0.6362	0.0053	0.0001

Table S11. P-values of the Wilcoxon rank test for the 150 best features vs. CV-optimized features

SG	L MsigDB	L KEGG	C NetC	C I2D
0.7375	0.0432	0.1109	0.1694	0.6733

3 Restricted gene sets are not detrimental to composite feature classifiers

Table S12. P-values of the Wilcoxon rank test between the unrestricted SG and each restricted SG with CV-optimized features. The set to which the single gene classifier is restricted when selecting genes, is depicted in the top row.

	HPRD	I2D	NetC	KEGG	MsigDB
unrestr	0.5372	0.0007	0.0837	0.0483	0.0460

Table S13. P-values of the Wilcoxon rank test between the unrestricted SG and each restricted SG with the best 50 features

	HPRD	I2D	NetC	KEGG	MsigDB
unrestr	0.3286	0.1682	0.7189	0.0069	0.1403

Table S14. P-values of the Wilcoxon rank test between the unrestricted SG and each restricted SG with the best 100 features

	HPRD	I2D	NetC	KEGG	MsigDB
unrestr	0.1981	0.3235	1.0000	0.1048	0.1977

Table S15. P-values of the Wilcoxon rank test between the unrestricted SG and each restricted SG with the best 150 features

	HPRD	I2D	NetC	KEGG	MsigDB
unrestr	0.3709	0.0270	0.6971	0.8936	0.3457

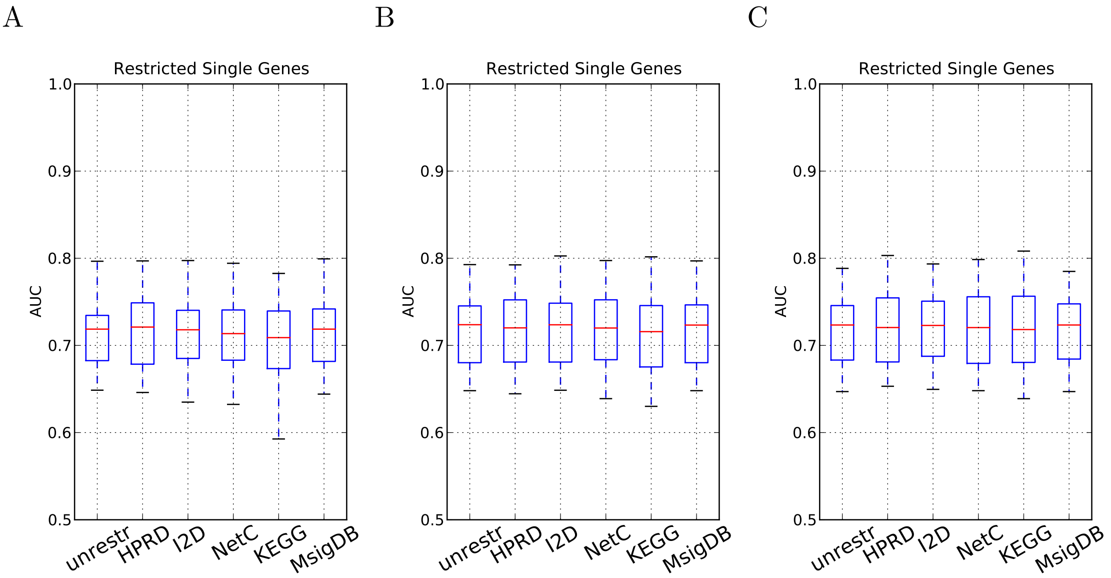

Figure S8. Restricted single-gene classifiers - fixed number of features. A: the best 50 single genes; **B:** the best 100 single genes; **C:** the best 150 single genes.

4 Training set size has no significant effect of performance differences

Affymetrix data - Paired setting

Table S16. P-values of the Wilcoxon rank test. Affymetrix data, paired setting, with CV-optimized features.

	L MsigDB	L KEGG	C KEGG	C NetC	C I2D	C HPRD
SG	0.5217	0.5503	0.1909	0.2455	0.c0153	0.4304

Table S17. P-values of the Wilcoxon rank test. Affymetrix data, paired setting, with the best 50 features.

	L MsigDB	L KEGG	C KEGG	C NetC	C I2D	C HPRD
SG	0.0897	0.4980	0.4813	0.0759	0.0023	0.2024

Table S18. P-values of the Wilcoxon rank test. Affymetrix data, paired setting, with the best 100 features.

	L MsigDB	L KEGG	C NetC	C I2D	C HPRD
SG	0.2162	0.9854	0.0136	0.0002	0.1054

Table S19. P-values of the Wilcoxon rank test. Affymetrix data, paired setting, with the best 150 features.

	L MsigDB	L KEGG	C NetC	C I2D	C HPRD
SG	0.0192	0.7012	0.0400	0.0006	0.2305

Affymetrix data, merged setting

Table S20. P-values of the Wilcoxon rank test. Affymetrix data, merged setting, with CV-optimized features.

	L MsigDB	L KEGG	C KEGG	C NetC	C I2D	C HPRD
SG	0.1875	0.1250	0.6250	0.1875	0.1875	0.1250

Table S21. P-values of the Wilcoxon rank test. Affymetrix data, merged setting, with the best 50 features.

	L MsigDB	L KEGG	C NetC	C I2D	C HPRD
SG	0.3125	0.1875	0.1250	0.3125	0.3125

Table S22. P-values of the Wilcoxon rank test. Affymetrix data, merged setting, with the best 100 features.

	L MsigDB	L KEGG	C NetC	C HPRD
SG	0.3125	0.3125	0.0625	0.3125

Table S23. P-values of the Wilcoxon rank test. Affymetrix data, merged setting, with the best 150 features.

	L MsigDB	L KEGG	C NetC	C HPRD
SG	0.3125	0.3125	0.1250	0.3125

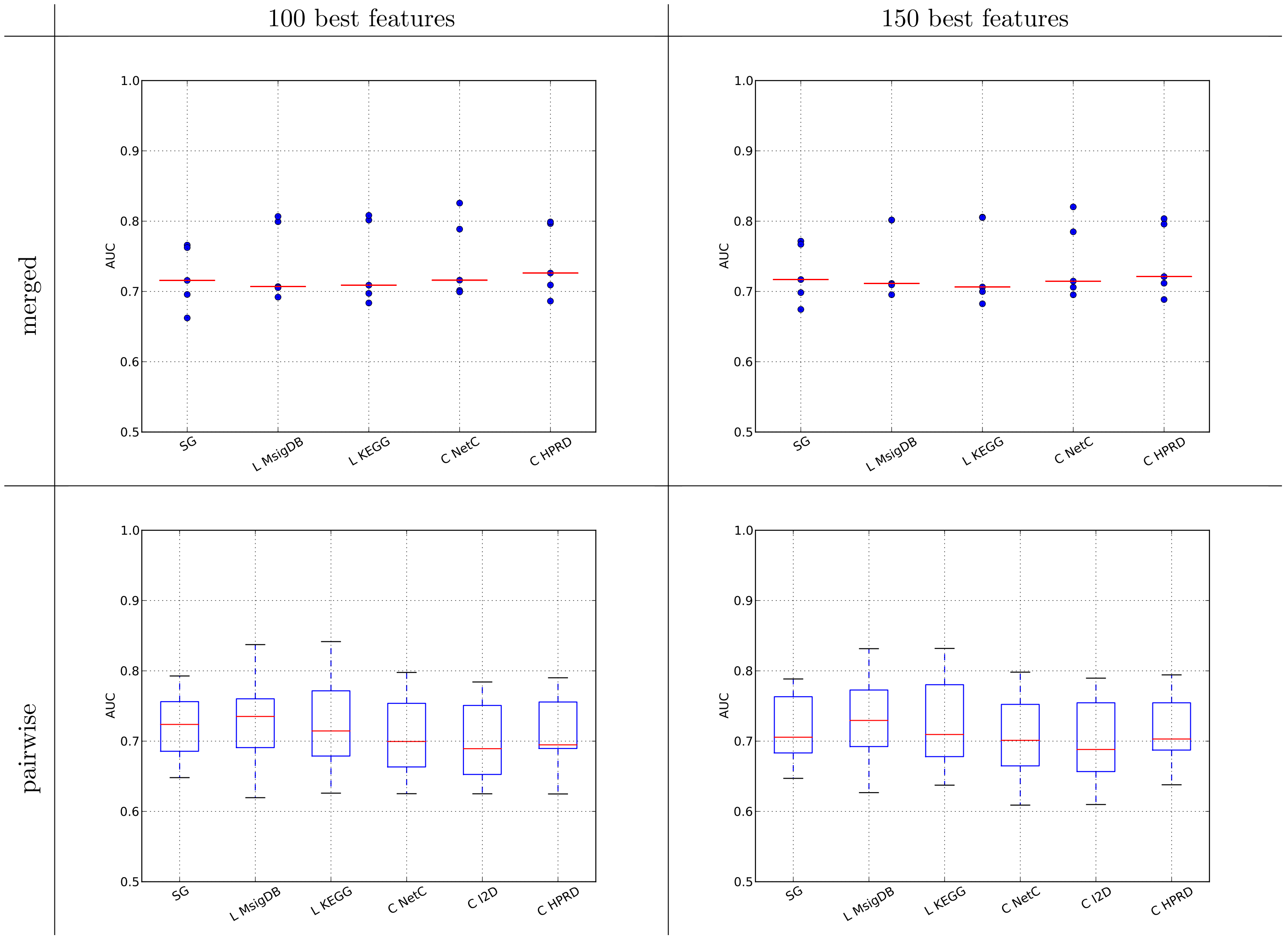

Figure S9. Classification results for the 'merged' and 'pairwise' setting. In the 'merged' setting one Affymetrix dataset is set aside as test and the remaining four Affymetrix dataset are merged into a single dataset. This is repeated until every one of the five datasets acted as a test set. **Top row:** Results for the merged setting. The red lines indicate the median. **Bottom row:** Only the five Affymetrix datasets were used in the pairwise setting.

5 Dataset homogeneity affects single genes and composite classifiers similarly

Table S24. P-values of the Wilcoxon rank test. ER positive data, 'merged-setting', with CV-optimized features.

	L MsigDB	L KEGG	C KEGG	C NetC	C I2D	C HPRD
SG	0.8438	1.0000	0.2807	0.8438	1.0000	0.8438

Table S25. P-values of the Wilcoxon rank test. ER positive data, 'merged-setting', with the 50 best features.

	L MsigDB	L KEGG	C NetC	C HPRD
SG	0.6875	1.0000	0.6875	0.5625

Table S26. P-values of the Wilcoxon rank test. ER positive data, 'merged-setting', with the 100 best features.

	L MsigDB	L KEGG	C NetC
SG	0.7874	1.0000	0.8438

Table S27. P-values of the Wilcoxon rank test. ER positive data, 'merged-setting', with the 150 best features.

	L MsigDB	L KEGG
SG	0.8438	0.8438

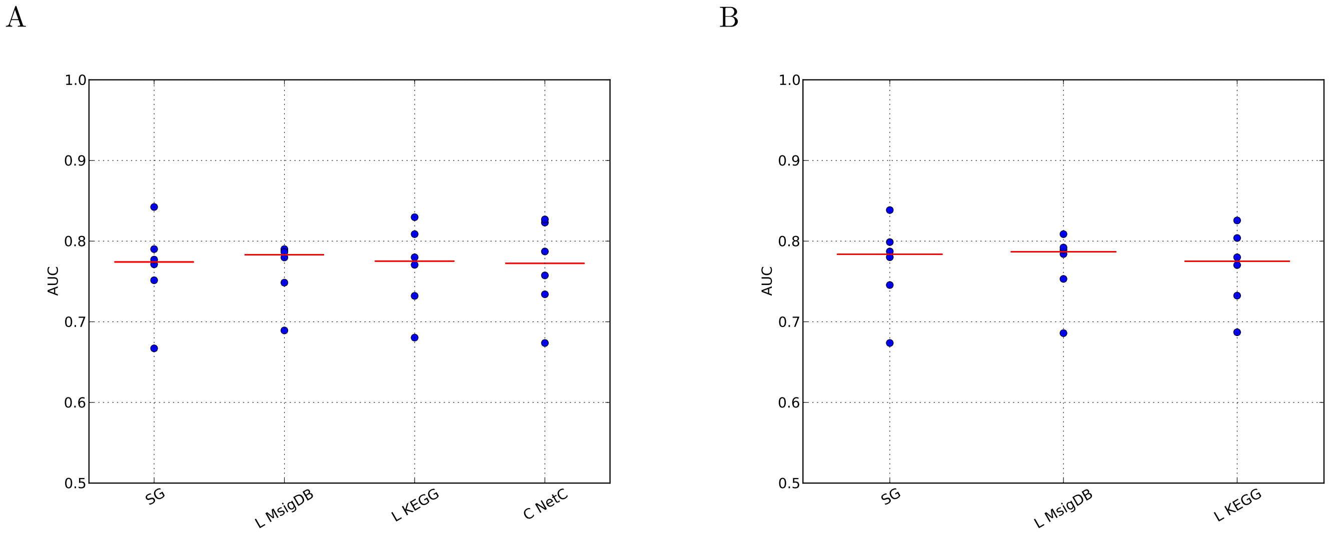

Figure S10. Classification results of the ER positive data for the merged setting, only using 100 and 150 features. A single dataset was set aside as test set while the remaining five datasets were merged into a single training set. This was repeated until each dataset was employed as left-out test set, resulting in six AUC values. **A**: 100 best features; **B**: 150 best features. The median is indicated as a red line.

6 Equal classification using real or randomized networks and pathways

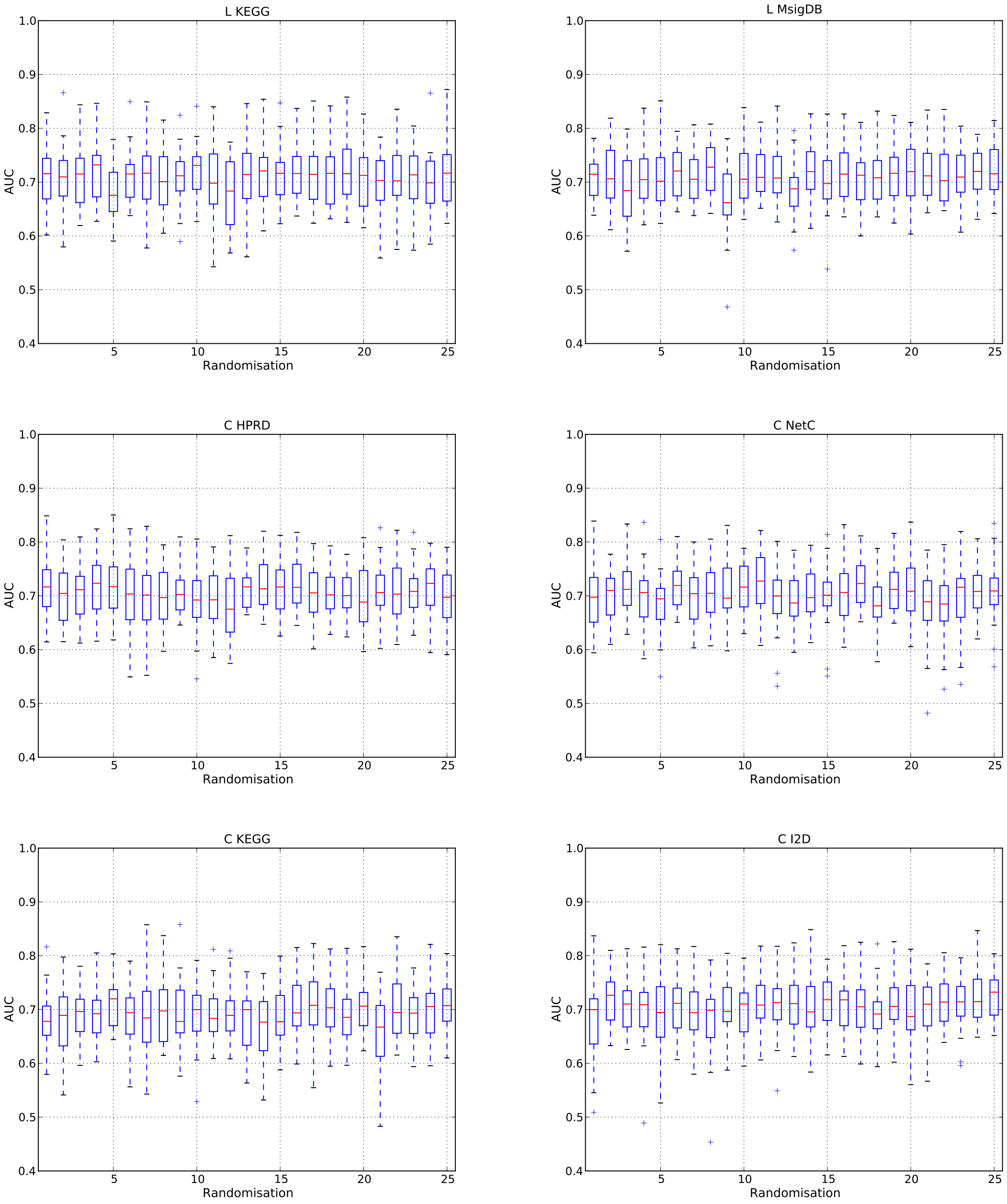

Figure S11. The effect of randomized secondary data sources. AUC values obtained with the feature extraction method *Lee* (L) on randomized KEGG and MsigDB pathways and AUC values obtained with the feature extraction method *Chuang* (C) on randomized PPI networks (KEGG, NetC, HPRD and OPHID). Shown are the AUC distributions for all 25 randomizations. For each randomization of the database we obtain 30 AUC values from the comparison procedure.

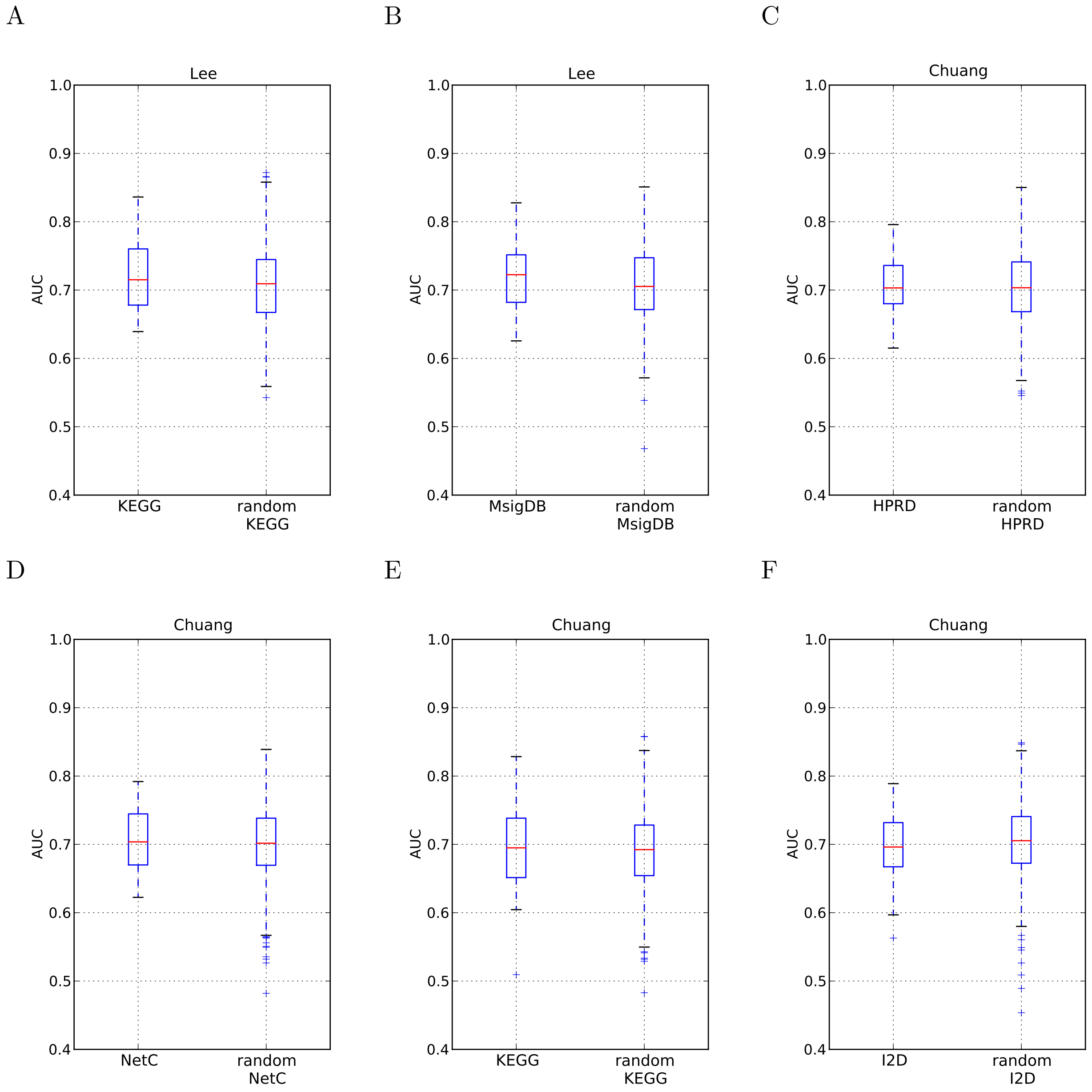

Figure S12. The effect of randomized secondary data sources. **A:** *Lee*-KEGG; **B:** *Lee*-MsigDB; **C:** *Chuang*-HPRD; **D:** *Chuang*-NetC; **E:** *Chuang*-KEGG; **F:** *Chuang*-I2D.

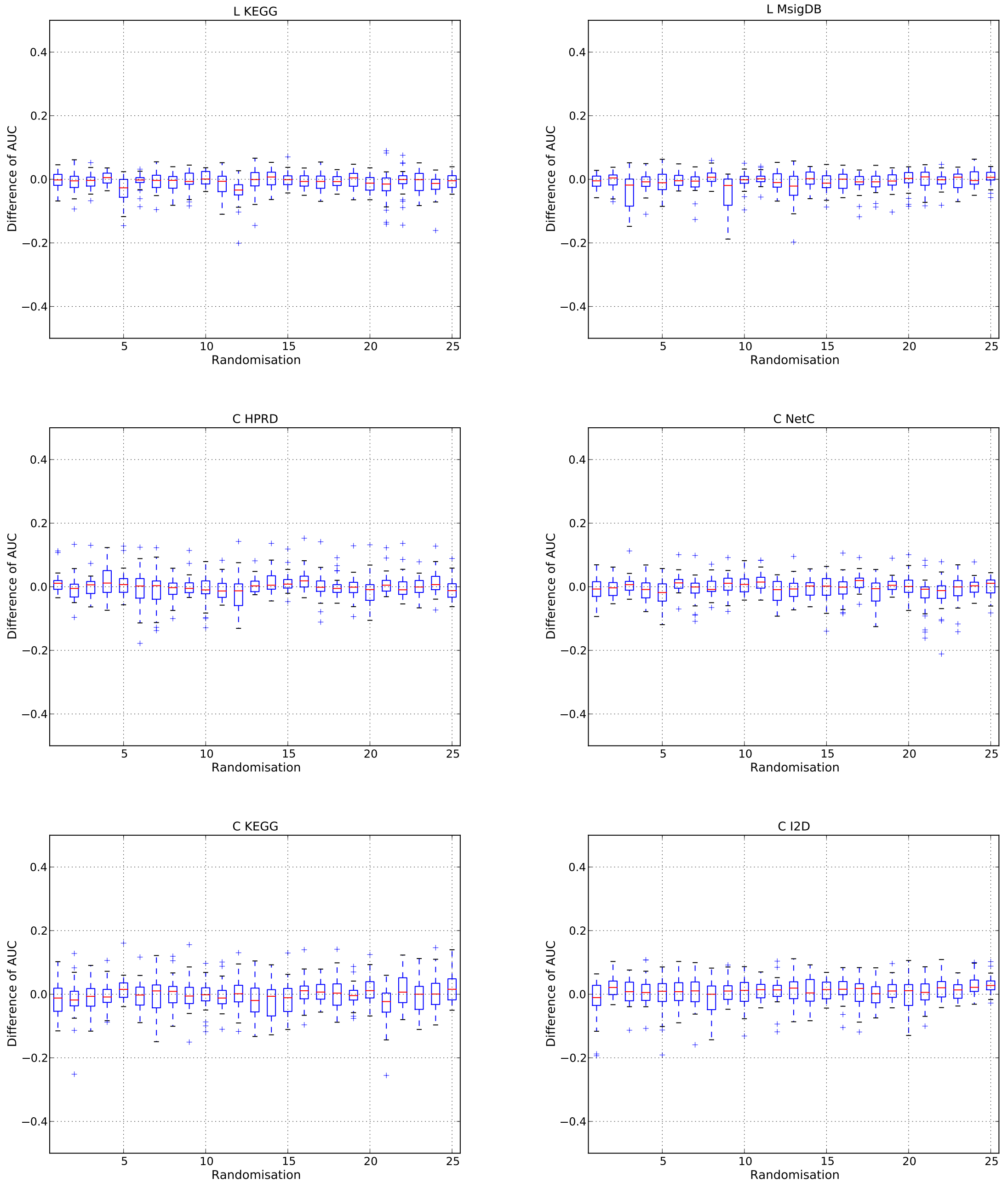

Figure S13. The difference in AUC values between the classifiers using randomized networks and the classifiers using the 'real' network. For each method the difference of the paired AUC values of the 'real distribution' and each 'random distribution' for each training-test dataset pair is shown. AUC values obtained with the feature extraction method *Lee* (L) on randomized KEGG and MsigDB pathways and AUC values obtained with the feature extraction method *Chuang* (C) on randomized PPI networks (KEGG, NetC, HPRD and OPHID). Shown are the AUC distributions for all 25 randomizations. For each randomization of the database we obtain 30 AUC values from the comparison procedure.

We calculated the one-sided paired Wilcoxon rank test between the ‘real’ AUC-value distribution and each of the 25 ’randomized’ AUC value distributions. The null hypothesis is that the real AUC distribution has a higher mean than the AUC distributions generated by employing randomized network and pathway data. We used Bonferroni correction to account for multiple testing. Tables S29-S33 show the p-values.

Table S28. P-values of the one-sided Wilcoxon rank test between the ‘real’ and the ‘random’ AUC distributions for *Lee* applied to KEGG

Randomization	1	2	3	4	5	6	7	8	9	10
	1.0000	1.0000	1.0000	1.0000	1.0000	1.0000	1.0000	1.0000	1.0000	1.0000
	11	12	13	14	15	16	17	18	19	20
	1.0000	1.0000	1.0000	1.0000	1.0000	1.0000	1.0000	1.0000	1.0000	1.0000
	21	22	23	24	25					
	1.0000	1.0000	1.0000	1.0000	1.0000					

Table S29. P-values of the Wilcoxon rank test between the ‘real’ and the ‘random’ AUC distributions for *Lee* applied to MsigDB

Randomization	1	2	3	4	5	6	7	8	9	10
	1.0000	1.0000	1.0000	1.0000	1.0000	1.0000	1.0000	1.0000	1.0000	1.0000
	11	12	13	14	15	16	17	18	19	20
	1.0000	1.0000	1.0000	1.0000	1.0000	1.0000	1.0000	1.0000	1.0000	1.0000
	21	22	23	24	25					
	1.0000	1.0000	1.0000	1.0000	1.0000					

Table S30. P-values of the Wilcoxon rank test between the ‘real’ and the ‘random’ AUC distributions for *Chuang* applied to HPRD

Randomization	1	2	3	4	5	6	7	8	9	10
	0.8737	1.0000	1.0000	1.0000	1.0000	1.0000	1.0000	1.0000	1.0000	1.0000
	11	12	13	14	15	16	17	18	19	20
	1.0000	1.0000	1.0000	0.9156	0.3666	0.0373	1.0000	1.0000	1.0000	1.0000
	21	22	23	24	25					
	1.0000	1.0000	1.0000	1.0000	1.0000					

Table S31. P-values of the Wilcoxon rank test between the 'real' and the 'random' AUC distributions for *Chuang* applied to NetC

Randomization	1	2	3	4	5	6	7	8	9	10
	1.0000	1.0000	1.0000	1.0000	1.0000	0.3621	1.0000	1.0000	1.0000	1.0000
	11	12	13	14	15	16	17	18	19	20
	0.2762	1.0000	1.0000	1.0000	1.0000	1.0000	0.0909	1.0000	1.0000	1.0000
	21	22	23	24	25					
	1.0000	1.0000	1.0000	1.0000	1.0000					

Table S32. P-values of the Wilcoxon rank test between the 'real' and the 'random' AUC distributions for *Chuang* applied to KEGG

Randomization	1	2	3	4	5	6	7	8	9	10
	1.0000	1.0000	1.0000	1.0000	0.1817	1.0000	1.0000	1.0000	1.0000	1.0000
	11	12	13	14	15	16	17	18	19	20
	1.0000	1.0000	1.0000	1.0000	1.0000	1.0000	1.0000	1.0000	1.0000	1.0000
	21	22	23	24	25					
	1.0000	1.0000	1.0000	1.0000	1.0000					

Table S33. P-values of the Wilcoxon rank test between the 'real' and the 'random' AUC distributions for *Chuang* applied to I2D

Randomization	1	2	3	4	5	6	7	8	9	10
	1.0000	0.0150	1.0000	1.0000	1.0000	1.0000	1.0000	1.0000	1.0000	1.0000
	11	12	13	14	15	16	17	18	19	20
	0.3242	0.5613	0.9156	1.0000	0.6647	0.1817	1.0000	1.0000	0.9590	1.0000
	21	22	23	24	25					
	1.0000	0.1241	0.7211	0.0015	0.0001					

Using real and randomized networks and pathways - results on the original data from Chuang et al. and Lee et al.

We employed the original breast cancer expression data (Wang and Vijver) and network and pathway data from the studies by Chuang *et al.* and Lee *et al.* We tested whether randomizing

the secondary data has any effect on the classification results. We only calculated the performances of the NMC using Wang as training dataset and Vijver as test dataset or vice versa. The expression datasets and the patients' class labels were provided by Chuang *et al.* as they were employed in their study. We found that the patients' class labels did not correspond to 5-year survival. Instead the censoring variable was used to stratify the patients without taking the time variable into account.

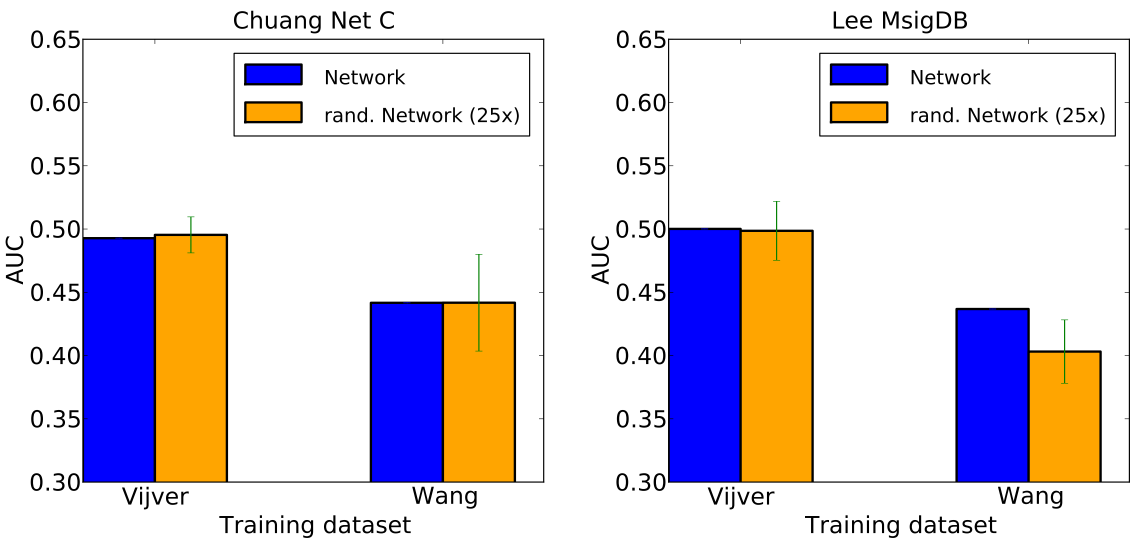

Figure S14. The effect of randomized secondary data sources. Left: AUC values obtained with the feature extraction method Chuang on the real NetC and randomized NetC. **Right:** AUC values obtained with the feature extraction method *Lee* on the real and randomized MsigDB pathways. In both cases we used the two original expression datasets and patient class labels as employed in Chuang *et al.* (Vijver and Wang). We employed one dataset as training dataset (indicated on the x-axis) and the other one as testing dataset. Apart from the combination *Lee*-MsigDB, training on Wang and testing on Vijver; there are no significant differences between the AUC values obtained when employing the original secondary data source and the AUC values obtained from 25 randomized secondary data sources. (Tested with a one-sample t-test.)

7 Current composite feature classifiers do not increase the stability of gene markers

7.1 The Fisher exact test as measure for overlap

A B

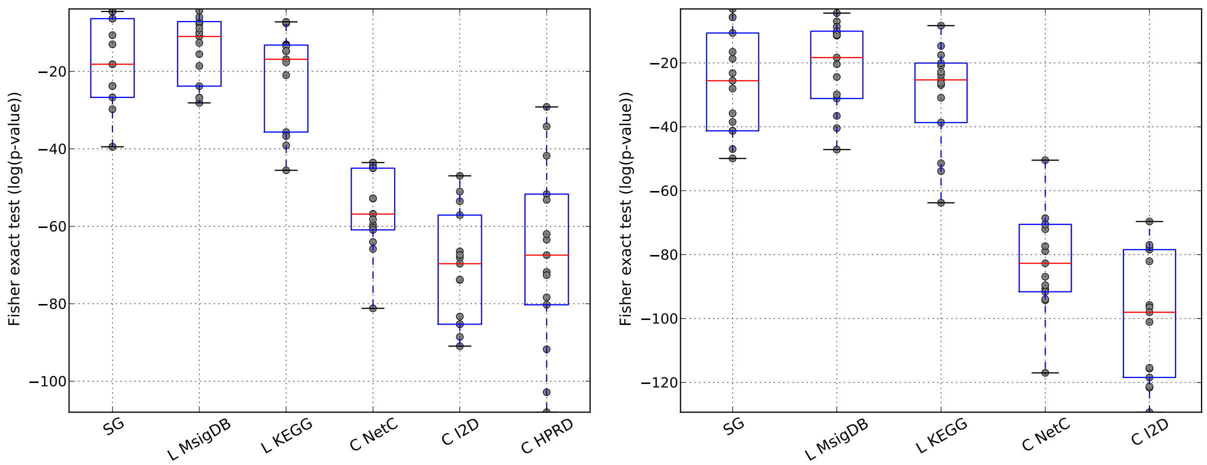

Figure S15. Feature stability when the top 100 and 150 features are selected. For each method the p-value of the Fisher exact test was calculated between the gene sets extracted from two different data sets. This was repeated for all pairwise combinations of data sets and these values are represented as a dotplot with the median indicated as a red line. Plotted are the log p-values. **A**: Feature stability when the top 100 features are selected. **B**: Feature stability when the top 150 features are selected.

Table S34. P-values of the Wilcoxon rank test. Single genes features overlap versus all network and pathway based features overlap across all pairs of datasets.

50 best features					
	L MsigDB	L KEGG	C NetC	C I2D	C HPRD
SG	0.9780	0.2293	0.0001	0.0001	0.0001
100 best features					
	L MsigDB	L KEGG	C NetC	C I2D	C HPRD
SG	0.5995	0.4887	0.0001	0.0001	0.0001
150 best features					
	L MsigDB	L KEGG	C NetC	C I2D	
SG	0.3894	0.5245	0.0001	0.0001	

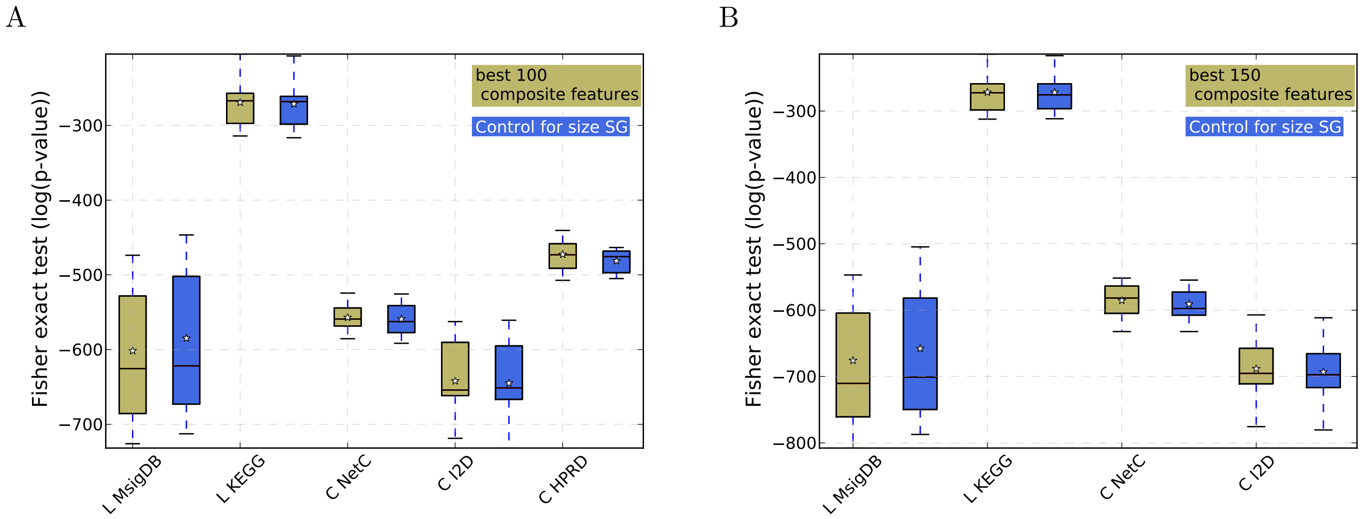

Figure S16. Feature stability when corrected for gene set size. Box plots of the Jaccard indices computed for all pairs of gene sets derived from two different data sets. The green box plots represent the Jaccard indices for genes constituting composite features, while the blue box plots (denoted as 'Control for size SG') represent the gene size corrected Jaccard indices for single-gene classifiers. The white stars represent the mean of the distributions. **A:** Feature stability for the 100 best composite features, **B:** Feature stability for the 150 best composite features.

We calculated the Wilcoxon rank test between the overlap of the genes in the composite features and the control for size single-gene markers.

Table S35. P-values of the Wilcoxon rank test between the genes in the 50 best composite features vs. control-for-size single-gene markers.

L MsigDB	L KEGG	C NetC	C I2D	C HPRD
0.0027	0.2769	0.3028	0.1354	0.8017

Table S36. P-values of the Wilcoxon rank test between the genes in the 100 best composite features vs. control-for-size single-gene markers.

L MsigDB	L KEGG	C NetC	C I2D	C HPRD
0.0002	0.0103	0.5245	0.3303	0.0034

Table S37. P-values of the Wilcoxon rank test between genes in the 150 best composite features vs. control-for-size single-gene markers.

L MsigDB	L KEGG	C NetC	C I2D
0.0002	0.5245	0.0328	0.0020

7.2 Overlap measured as Jaccard index

In addition to the Fisher exact test, we calculated the overlap between gene marker sets by employing the Jaccard index. As in the main document, we also correct the size of the single genes sets. For each data set and each feature selection approach employing secondary data sources, we obtain a single best feature set consisting of n^* features (networks, gene sets or pathways) where each feature, in turn, consists of m genes. We then determine a size-matched single gene set by choosing the best m single genes on that same expression data set.

A factor that influences the Jaccard index is the size of the starting set of genes from which marker gene sets are chosen. In case of the single genes method, markers can be chosen from the whole array, i.e. 11601 genes whereas in the case of the network and pathway based methods only genes that are annotated in the specific secondary data source can be chosen. For this reason we used a random subsample of the same size as the secondary data source as starting set for the single genes. Assuming a secondary data source that contains N annotated genes, then we proceeded as described in Algorithm 1 to determine the 'control for size' single gene markers.

Algorithm 1 Select the control for size single genes markers

```
for R = 1  to  100 do
    Randomly select N genes, call this set R
    /* Iterate over all datasets d_i */
    for i ∈ [d_1, d_2, ..., d_M] do
        Rank all genes in R based on d_i by their t-statistic from best to worst, call this ranked
          list L_i
        Determine the number of genes in the top 50 networks for i, denote that by N_i
        Select the top N_i genes in L_i, denote this by S_i
    end for
    Determine the Jaccard indices between all pairs of S_i, i < j denoted by J_ijR, where R
      represents the randomization
end for
Calculate for each dataset pair the average over the 100 values of J_ijR : J̄_ij = (Σ_R J_ijR) / 100
```

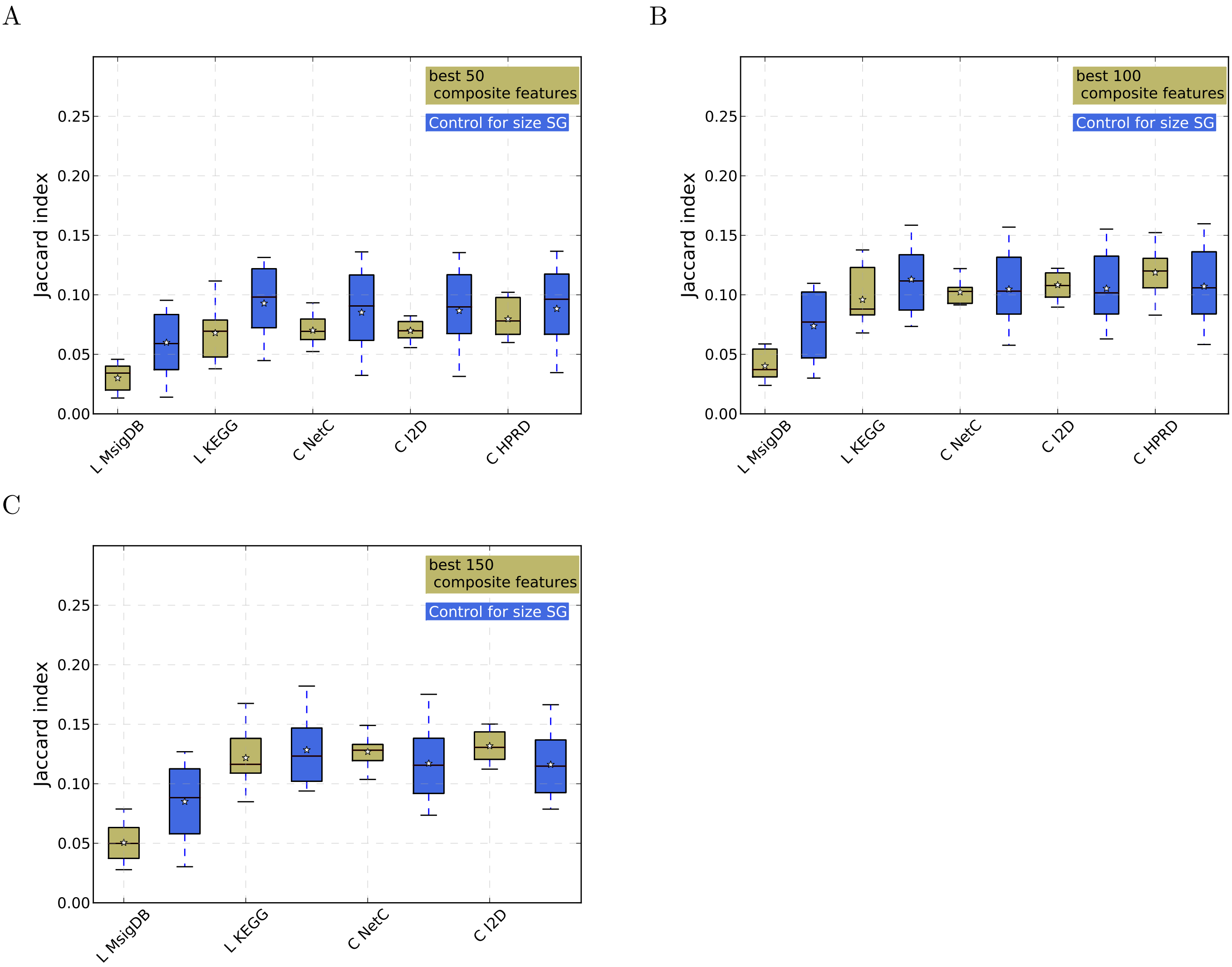

Figure S17. Overlap of the network gene markers across the six datasets vs. the mean Jaccard index of the control for size single genes sets across the six datasets. A: 50 best features; B: 100 best features and C: 150 best features

Table S38. P-values of the Wilcoxon rank test between the genes in the 50 best composite features vs. control-for-size single-gene markers drawn from random subsampling.

L MsigDB	L KEGG	C NetC	C I2D	C HPRD
0.0012	0.0020	0.0946	0.0413	0.3028

Table S39. P-values of the Wilcoxon rank test between the genes in the 100 best composite features vs. control-for-size single-gene markers drawn from random subsampling.

L MsigDB	L KEGG	C NetC	C I2D	C HPRD
0.0002	0.0103	0.8904	0.8469	0.1688

Table S40. P-values of the Wilcoxon rank test between genes in the 150 best composite features vs. control-for-size single-gene markers drawn from random subsampling.

L MsigDB	L KEGG	C NetC	C I2D
0.0002	0.1205	0.3303	0.0302

8 PinnacleZ

Concerning the implementation of the algorithm by Chuang *et al.* above, we found that PinnacleZ is not identical to the original implementation of the authors. Given the same input parameters than in the original study, PinnacleZ usually identifies a larger number of significant subnetworks.

The results returned by PinnacleZ are not reproducible. To calculate the null distributions for the three statistical tests the implementation employs a random generator without fixing the seed. Furthermore, it uses a single random generator from within multiple threads, which makes the code non-deterministic even if one initializes the seed to a known value. This means that PinnacleZ returns different outputs on identical runs.

Unfortunately, the original code for the network search and the cross validation used in Chuang *et al.* was no longer available.
